# Targeting β-Adrenergic Signaling in Colorectal Cancer: Molecular Mechanisms and Therapeutic Potential of β-Blockers

**DOI:** 10.3390/cancers18152507

**Published:** 2026-08-05

**Authors:** Zuzanna Rogacz, Wiktoria Weronika Pacuła, Wiktor Janas, Magda Markiewka, Paulina Wala, Marcel Madej, Barbara Strzałka-Mrozik

**Affiliations:** 1 Department of Molecular Biology, Faculty of Pharmaceutical Sciences in Sosnowiec, Medical University of Silesia, 40-055 Katowice, Poland; s87155@365.sum.edu.pl (Z.R.); s87164@365.sum.edu.pl (W.W.P.); s87157@365.sum.edu.pl (W.J.); s87161@365.sum.edu.pl (M.M.); marcel.madej@sum.edu.pl (M.M.); 2 Silesia LabMed: Centre for Research and Implementation, Medical University of Silesia, 40-752 Katowice, Poland; paulina.wala@sum.edu.pl

**Keywords:** colorectal cancer, beta-blockers, drug repurposing, propranolol, β-adrenergic signaling, tumor microenvironment, angiogenesis, apoptosis

## Abstract

Colorectal cancer (CRC) remains one of the leading causes of cancer-related morbidity and mortality worldwide despite substantial advances in surgery, chemotherapy, targeted therapies, and immunotherapy. The limited efficacy of current treatment strategies in advanced disease and the emergence of therapeutic resistance highlight the urgent need for novel adjunctive therapeutic approaches. Increasing evidence indicates that chronic stress and sustained activation of β-adrenergic signaling promote colorectal tumor initiation, progression, angiogenesis, metastatic dissemination, and immune evasion, thereby identifying this pathway as a potential therapeutic target. Drug repurposing has emerged as an attractive strategy for accelerating the development of new anticancer therapies by identifying novel applications for clinically approved drugs with well-established safety profiles. Among these, β-blockers have gained considerable attention because of their ability to inhibit β-adrenergic signaling and modulate multiple oncogenic pathways implicated in CRC progression. Although accumulating preclinical and observational clinical evidence suggests that β-blockers may possess anticancer potential, the underlying molecular mechanisms and their translational relevance have not yet been comprehensively integrated. This review provides a critical overview of the current evidence regarding the therapeutic potential of β-blockers in CRC by integrating findings from preclinical and clinical studies. Particular emphasis is placed on the regulation of key signaling pathways, including cAMP/PKA/CREB, PI3K/AKT/mTOR, and RAS/RAF/MEK/ERK, as well as on the effects of β-blockers on tumor cell proliferation, apoptosis, angiogenesis, epithelial–mesenchymal transition, metastasis, and modulation of the tumor microenvironment and antitumor immune responses. However, significant barriers limit the translation of these findings into routine clinical practice, including the limited representativeness of preclinical models, potential hemodynamic adverse effects, and the inherent limitations of observational studies. Importantly, owing to the lack of prospective randomized clinical trials, the current evidence remains insufficient to establish the clinical efficacy of β-blockers in CRC. Although β-blockers possess several characteristics that make them attractive candidates for drug repurposing, including a well-established safety profile, widespread availability, and low cost, further mechanistic studies, prospective randomized clinical trials, and biomarker-based patient stratification are essential to determine their clinical efficacy and define their role in personalized CRC therapy.

## Simple Summary

Colorectal cancer (CRC) remains one of the leading causes of cancer-related death worldwide despite continuous advances in diagnosis and treatment. Increasing evidence suggests that chronic stress promotes CRC progression through activation of β-adrenergic signaling, making this pathway an attractive therapeutic target. Because developing new anticancer drugs is costly and time-consuming, repurposing clinically approved medications has become an increasingly important strategy. This review summarizes and critically discusses current evidence supporting the use of β-blockers, particularly propranolol, as potential adjunctive agents in CRC therapy. We describe how β-blockers may inhibit tumor growth, angiogenesis, metastasis, and immune evasion by modulating multiple molecular signaling pathways and the tumor microenvironment. We also discuss the current limitations of available evidence and future directions for biomarker-guided clinical studies. Given their well-established safety profile, widespread clinical use, and low cost, β-blockers represent promising candidates for future personalized treatment strategies in CRC.

## 1. Introduction

Colorectal cancer (CRC) remains one of the leading causes of cancer-related morbidity and mortality worldwide. It is the third most frequently diagnosed malignancy and the second leading cause of cancer-related death, accounting for approximately 2.04 million new cases and 917,895 deaths globally, representing 9.9% of all cancer diagnoses and 9.4% of all cancer-related deaths. Despite substantial advances in prevention, early detection, surgical techniques, systemic therapies, and immunotherapy, CRC continues to impose a considerable global health burden. The disease is more common in men than in women and exhibits marked geographic variation, with the highest incidence rates reported in Europe, Australia and New Zealand, and Northern America [1,2,3,4]. Epidemiological projections indicate that the global burden of CRC will continue to rise over the coming decades, driven by population aging, urbanization, obesity, sedentary lifestyle, and dietary changes [1,5,6,7]. These trends underscore the urgent need to identify novel therapeutic strategies that complement existing treatment modalities and improve clinical outcomes, particularly for patients with advanced or treatment-resistant disease.

CRC is a highly heterogeneous malignancy that arises through a complex interplay of genetic, epigenetic, environmental, and lifestyle-related factors [8]. Its development is characterized by progressive accumulation of molecular alterations that disrupt the regulation of cell proliferation, differentiation, apoptosis, invasion, and metastatic dissemination [1,8]. Among the most common genetic events are mutations in the adenomatous polyposis coli (*APC*), *KRAS*, *NRAS*, *BRAF*, *TP53*, and *PIK3CA* genes, leading to dysregulation of key oncogenic signaling pathways, including Wnt/β-catenin, epidermal growth factor receptor (EGFR), and PI3K/AKT [1]. Tumor progression is further driven by chromosomal instability (CIN), microsatellite instability (MSI), deficient mismatch repair (dMMR), and the CpG island methylator phenotype (CIMP), all of which contribute to the marked biological heterogeneity of CRC and profoundly influence prognosis, therapeutic response, and clinical outcomes [1,8,9,10].

This molecular heterogeneity is further reflected in the Consensus Molecular Subtypes (CMS1–CMS4), which differ in their molecular characteristics, tumor microenvironment (TME) composition, prognosis, and response to therapy. CMS1 is associated with MSI/dMMR and immune activation, CMS2 with canonical WNT/MYC signaling, CMS3 with metabolic dysregulation and frequent KRAS mutations, whereas CMS4 exhibits mesenchymal features, stromal activation, and poor prognosis. In addition, clinically relevant biomarkers, including KRAS, NRAS, BRAF^V600E^, MSI/dMMR, and human epidermal growth factor receptor 2 (HER2), play central roles in patient stratification and therapeutic decision-making by predicting responses to anti-EGFR therapy, immune checkpoint inhibitors, and other targeted therapies [1]. Although the influence of these molecular characteristics on the therapeutic efficacy of β-blockers remains unknown, they should be considered in future biomarker-guided studies evaluating β-blocker repurposing in CRC. Current CRC management relies on a multidisciplinary approach combining surgery, chemotherapy, radiotherapy, targeted therapies, and immunotherapy. Although these treatment modalities have substantially improved patient survival, their long-term efficacy remains limited by treatment-related toxicity, marked tumor heterogeneity, and the development of intrinsic or acquired therapeutic resistance. Consequently, many patients ultimately experience disease progression and metastatic dissemination despite receiving standard-of-care treatment [9,11,12]. These limitations highlight the urgent need for novel therapeutic strategies capable of enhancing treatment efficacy while minimizing toxicity and overcoming resistance.

Chronic psychological stress is increasingly recognized as an important contributor to CRC progression because it represents one of the principal physiological triggers of sustained sympathetic nervous system activation and β-adrenergic signaling. Persistent catecholamine release activates β-adrenergic receptors (β-ARs) expressed by both tumor cells and cells within the TME, providing the biological rationale for investigating β-blockers as modulators of these pathways in CRC.

Activation of β-adrenergic signaling has been implicated in multiple hallmarks of cancer. Adrenaline (epinephrine) and noradrenaline (norepinephrine), the principal mediators of the stress response, exert their biological effects through α- and β-ARs expressed not only by tumor cells but also by stromal, endothelial, and immune cells within the TME. Sustained β-adrenergic signaling promotes tumor cell proliferation, angiogenesis, epithelial–mesenchymal transition (EMT), metastatic dissemination, metabolic adaptation, and suppression of antitumor immune responses, thereby contributing to CRC progression [13,14,15].

Drug repurposing has emerged as a promising strategy for accelerating the development of novel anticancer therapies by identifying new oncological applications for clinically approved drugs with well-established pharmacological and safety profiles. Among these agents, β-blockers have attracted increasing attention because of their ability to inhibit β-adrenergic signaling, a pathway now recognized as a key regulator of tumor progression and tumor–host interactions [14,15,16]. Accumulating experimental and clinical evidence indicates that β-ARs are widely expressed in CRC cells as well as in multiple cellular components of the TME, suggesting that their pharmacological blockade may exert pleiotropic antitumor effects extending beyond cardiovascular disease management. Nevertheless, despite the growing interest in β-blockers as repurposed anticancer agents, the available evidence remains fragmented and several important questions remain unresolved. Previous reviews have examined this field from complementary but distinct perspectives. Recent publications have primarily discussed β-blockers as adjuncts to conventional therapies and immunotherapy across multiple malignancies, whereas Ghosn et al. provided a CRC-specific overview of β-adrenergic signaling, associated risk factors, and the available preclinical and clinical evidence [15,16]. More recently, Varghese et al. [17] focused on the β_2_-AR-mediated neuro-neoplastic axis in CRC, while Jiang et al. [18] reviewed adrenergic signaling within the broader context of neurotransmitter-mediated colorectal carcinogenesis and drug repurposing. Despite these important contributions, several important gaps remain. Previous reviews have not comprehensively integrated β-blocker pharmacology and receptor selectivity with the molecular mechanisms underlying β-adrenergic signaling, including the cAMP/PKA/CREB, PI3K/AKT/mTOR, and RAS/RAF/MEK/ERK pathways, nor have they critically examined the effects of β-blockers on TME remodeling, angiogenesis, EMT, apoptosis, antitumor immunity, and treatment sensitization within a single CRC-specific framework. Furthermore, the considerable heterogeneity in β-AR expression, β-blocker selectivity, experimental models, and clinical study design complicates interpretation of the available evidence and limits its clinical translation. Moreover, substantial heterogeneity in β-AR expression, β-blocker selectivity, and study design complicates interpretation of the available evidence and limits its clinical translation. Consequently, a comprehensive evaluation of the molecular rationale, preclinical evidence, clinical data, and future therapeutic perspectives is needed to better define the role of β-blockers in CRC management.

Therefore, this review provides a comprehensive and critical overview of the role of β-adrenergic signaling in CRC and evaluates the therapeutic potential of β-blockers as repurposed anticancer agents. Building upon previous reviews, it offers an integrated, CRC-specific perspective that combines current knowledge on β-blocker pharmacology and receptor selectivity with the molecular mechanisms of β-adrenergic signaling, including the cAMP/PKA/CREB, PI3K/AKT/mTOR, and RAS/RAF/MEK/ERK pathways. In addition, the review considers the molecular heterogeneity of CRC, including the CMS1–CMS4 and clinically relevant biomarkers, to place the available evidence within the context of precision oncology. Furthermore, it comprehensively discusses the effects of β-blockers on TME remodeling, angiogenesis, EMT, apoptosis, immune regulation, and treatment sensitization by critically integrating the available preclinical and clinical evidence within a unified translational framework. Particular emphasis is placed on the current limitations of β-blocker repurposing, barriers to clinical implementation, biomarker-guided patient stratification, and future research priorities, including the need for well-designed prospective randomized clinical trials to establish the clinical efficacy of β-blockers in CRC.

## 2. Current Treatment of Colorectal Cancer

### 2.1. Surgical Treatment

Surgery remains the cornerstone of treatment for patients with localized and resectable CRC. Current surgical management includes conventional open surgery as well as minimally invasive laparoscopic and robotic-assisted techniques, which are associated with reduced surgical trauma, faster postoperative recovery, and improved perioperative outcomes [19,20,21,22]. In selected patients, endoscopic resection may be performed for premalignant lesions and early-stage tumors, whereas local ablative procedures can be considered for carefully selected metastatic liver or lung lesions [19,20,23,24].

Despite substantial advances in surgical techniques, postoperative complications, including anastomotic leakage, infections, bowel obstruction, and thromboembolic events, continue to adversely affect patient outcomes by increasing morbidity, mortality, and healthcare utilization [20]. Furthermore, surgery alone is frequently insufficient to prevent disease recurrence, particularly in patients with locally advanced or metastatic colorectal cancer (mCRC). Consequently, optimal patient management requires a multidisciplinary approach integrating surgery with systemic therapies and long-term surveillance to improve both survival and quality of life [20,25,26].

### 2.2. Chemotherapy

Chemotherapy remains a cornerstone of CRC treatment, particularly in patients with locally advanced, metastatic, or high-risk disease. Current therapeutic strategies are based on fluoropyrimidines, including 5-fluorouracil (5-FU) and capecitabine (CAP), administered either as monotherapy or in combination with oxaliplatin and/or irinotecan. Commonly used regimens include FOLFOX, FOLFIRI, CAPOX, XELIRI, and, in selected patients, the more intensive FOLFOXIRI protocol [27,28,29,30].

Chemotherapy plays an important role in both perioperative and metastatic settings. Adjuvant treatment is routinely administered following curative surgery in patients with stage III CRC and selected cases of high-risk stage II disease to reduce recurrence risk and eradicate residual micrometastatic disease. Neoadjuvant chemotherapy may also be used in selected patients to reduce tumor burden, improve resectability, and facilitate subsequent surgical intervention [21,23,31,32,33].

Despite substantial improvements in patient outcomes, chemotherapy remains limited by systemic toxicity, treatment-related adverse effects, and the development of intrinsic or acquired drug resistance, all of which compromise long-term therapeutic efficacy. Common toxicities, including myelosuppression, peripheral neuropathy, gastrointestinal disturbances, and fatigue, frequently require dose reductions or treatment discontinuation, thereby negatively affecting both treatment effectiveness and patients’ quality of life [20,27]. Furthermore, disease recurrence and resistance to fluoropyrimidine- and oxaliplatin-based regimens remain major clinical challenges. These limitations underscore the need for complementary therapeutic strategies capable of enhancing treatment efficacy while minimizing toxicity, providing a strong rationale for investigating drug-repurposing approaches, including β-blockers, as potential adjunctive therapies.

### 2.3. Radiotherapy

Radiotherapy is an important component of CRC treatment, particularly for patients with locally advanced rectal cancer at increased risk of local recurrence. In the neoadjuvant setting, it induces deoxyribonucleic acid (DNA) damage, promotes tumor cell death, facilitates tumor downstaging, improves resectability, and reduces the risk of local recurrence [19,32,34,35]. In selected clinical settings, radiotherapy may also provide effective local control or palliation of metastatic lesions [19,34].

Despite its established clinical benefits, radiotherapy remains associated with important limitations, including damage to surrounding healthy tissues, acute and late gastrointestinal and hematological toxicities, infertility, sexual dysfunction, and the risk of radiation-induced secondary malignancies. Furthermore, its therapeutic efficacy is constrained by normal tissue dose limitations, radioresistance, and disease recurrence outside the irradiated field. Consequently, individualized treatment planning and careful patient selection remain essential to maximize therapeutic benefit while minimizing toxicity [19,20]. These limitations further support the need for complementary therapeutic approaches that enhance treatment efficacy without increasing treatment-related toxicity.

### 2.4. Targeted Therapies

Targeted therapies have become an integral component of the management of mCRC, with treatment selection guided by molecular biomarkers, including *RAS* (KRAS/NRAS), *BRAF^V600E^*, *HER2* amplification, and microsatellite instability/deficient mismatch repair (MSI-H/dMMR) status [27,36]. Current treatment algorithms incorporate biomarker-guided targeted therapies to selectively inhibit key molecular pathways involved in tumor growth, angiogenesis, survival, and therapeutic resistance.

For patients with *RAS* wild-type tumors, anti-EGFR monoclonal antibodies, including cetuximab and panitumumab, are recommended in combination with chemotherapy. Anti-vascular endothelial growth factor (VEGF) therapy, most commonly bevacizumab, remains a standard component of treatment across multiple molecular subgroups. Patients with *BRAF^V600E^*-mutated mCRC may benefit from combined encorafenib plus cetuximab, whereas HER2-positive tumors can be treated with *HER2*-targeted regimens, including combinations of trastuzumab with tucatinib or pertuzumab, following disease progression. In later treatment lines, additional targeted agents such as regorafenib and fruquintinib have expanded therapeutic options for selected patients [36,37,38,39].

Although targeted therapies have significantly improved clinical outcomes, their efficacy remains limited by intrinsic and acquired resistance, treatment-related toxicities, and the requirement for accurate molecular biomarker testing and continuous disease monitoring. Furthermore, the benefits of these agents are largely restricted to relatively small molecularly defined patient subgroups, while treatment costs and evolving resistance mechanisms continue to present important clinical challenges. Consequently, the identification of novel therapeutic targets and complementary therapeutic strategies remains essential for further improving outcomes in patients with CRC [27,36,37,38,39].

### 2.5. Immunotherapy

Immunotherapy has become an integral component of the treatment of molecularly selected patients with CRC, particularly those with metastatic MSI-H/dMMR tumors. Current treatment algorithms recommend immune checkpoint inhibitor (ICI)-based therapy as the standard first-line treatment for this patient population, whereas most microsatellite stable/proficient mismatch repair (MSS/pMMR) tumors remain largely refractory to immunotherapy [20,40,41,42].

ICIs targeting the programmed cell death protein 1 (PD-1)/programmed death-ligand 1(PD-L1) and cytotoxic T-lymphocyte-associated protein 4 (CTLA-4) pathways, including pembrolizumab, nivolumab, and the combination of nivolumab plus ipilimumab, restore antitumor T-cell activity and have demonstrated durable clinical benefit in patients with MSI-H/dMMR mCRC. In contrast, the efficacy of immunotherapy in MSS/pMMR tumors remains limited because of low immunogenicity and an immunosuppressive TME. Consequently, numerous combination strategies integrating ICIs with chemotherapy, targeted therapies, antiangiogenic agents, or other immunomodulatory approaches are currently under clinical investigation to improve treatment responses in this larger patient population [40,41,42].

Additional immunotherapeutic strategies, including adoptive cell therapies such as tumor-infiltrating lymphocytes (TILs), T-cell receptor-engineered T cells (TCR-T), chimeric antigen receptor T cells (CAR-T), CAR-natural killer (CAR-NK) cells, and therapeutic cancer vaccines, have shown encouraging preclinical and early clinical results but have not yet been incorporated into routine clinical practice [40,41,43,44,45].

Despite its remarkable clinical success in MSI-H/dMMR CRC, immunotherapy remains limited by the relatively small proportion of patients who derive durable benefit, primary and acquired resistance, immune-related adverse events, and the lack of reliable predictive biomarkers beyond MSI/dMMR status. Therefore, the development of novel biomarkers and rational combination strategies remains essential to expand the clinical benefit of immunotherapy to a broader population of patients with CRC [20,40,41,42,43,44,45].

Overall, effective CRC management requires a personalized, multidisciplinary approach integrating surgery, chemotherapy, radiotherapy, targeted therapies, and immunotherapy according to tumor stage, molecular profile, and patient-specific characteristics. Nevertheless, intrinsic and acquired therapeutic resistance, disease recurrence, treatment-related toxicities, and the limited applicability of biomarker-driven therapies continue to compromise long-term clinical outcomes. These challenges underscore the need for complementary therapeutic strategies. In this context, drug repurposing has emerged as a promising approach, with β-blockers representing potential adjunctive agents that may enhance the efficacy of existing therapies through modulation of tumor-promoting adrenergic signaling. A comparative summary of current treatment modalities is presented in Table 1.

## 3. Mechanisms of Action of β-Blockers

### 3.1. Structural Classification and Functional Diversity of Adrenergic Receptors and Their Modulation by β-Blockers

Adrenergic receptors (ARs) are a family of G protein-coupled receptors (GPCRs) that play a central role in mediating physiological responses to endogenous catecholamines, including epinephrine (Epi) and norepinephrine (NE), whose levels are elevated under conditions of chronic stress [15,46]. ARs are broadly classified into two major groups: α-adrenergic receptors and β-AR. Within the β-AR family, three principal subtypes have been identified: β_1_-AR, β_2_-AR, and β_3_-AR, each characterized by distinct tissue distribution and physiological functions [15,46].

The β_1_-AR subtype is predominantly expressed in cardiac tissue and the kidneys, where it regulates heart rate, myocardial contractility, and renin secretion. In contrast, β_2_-ARs are highly expressed in the lungs and vascular smooth muscle, where they mediate bronchodilation and vasodilation. The β_3_-AR subtype is primarily associated with adipose tissue metabolism, particularly lipolysis, and contributes to smooth muscle relaxation in the urinary bladder and gastrointestinal tract [46,47,48].

Under physiological conditions, activation of β-ARs by catecholamines initiates complex intracellular signaling pathways involved in the regulation of cardiovascular function, metabolism, smooth muscle tone, and adaptive responses to stress [46]. However, chronic activation of the adrenergic system has also been implicated in the progression of several malignancies, including CRC [47,49].

β-Blockers are a class of drugs that exert their pharmacological effects by selectively or non-selectively inhibiting β-AR activity. By preventing catecholamine binding, these agents suppress the activation of adrenergic signaling pathways [46]. Cardioselective β-blockers, such as bisoprolol, metoprolol, and nebivolol, preferentially inhibit β_1_-ARs and are widely used in cardiovascular medicine. In contrast, non-selective β-blockers, including propranolol (PrOH) and sotalol, inhibit both β_1_- and β_2_-ARs and have attracted considerable interest in oncology research. A third group comprises agents with additional α_1_-adrenergic receptor antagonistic activity, such as carvedilol and labetalol, which combine β-receptor blockade with vasodilatory effects [48,50].

Of particular relevance to cancer biology is the β_2_-AR subtype, which is expressed in both tumor cells and components of the TME. Increasing evidence indicates that β_2_-adrenergic signaling contributes to tumor growth, angiogenesis, EMT, metastatic dissemination, and suppression of antitumor immune responses. Consequently, pharmacological inhibition of β-adrenergic signaling by β-blockers has emerged as a promising therapeutic strategy in CRC and other solid malignancies [47,48].

**Figure 1:**
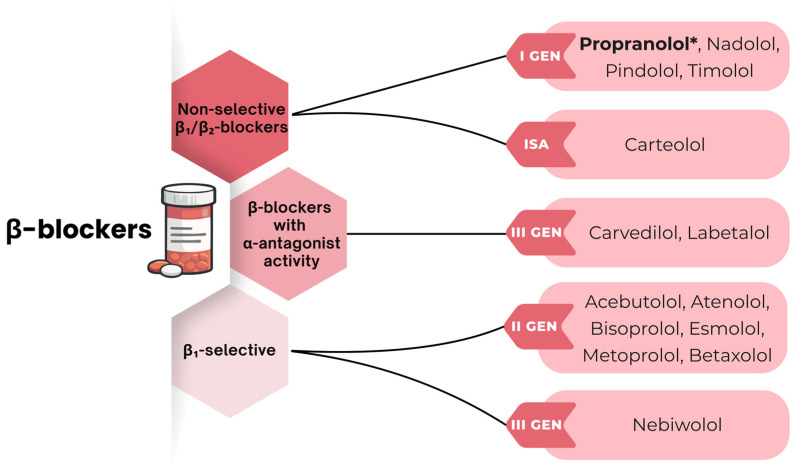
Classification of β-blockers according to receptor selectivity and pharmacological properties. [48,50,51]. Propranolol* is highlighted because it is the most extensively investigated non-selective β-blocker in colorectal cancer research. As the strongest body of preclinical and clinical evidence currently concerns propranolol, it serves as the principal reference compound throughout this review. **Abbreviations:** α—alpha-adrenergic receptor; α_1_—alpha-1 adrenergic receptor; β—beta-adrenergic receptor; β_1_—beta-1 adrenergic receptor; β_2_—beta-2 adrenergic receptor; β_1_/β_2_—beta-1 and beta-2 adrenergic receptor blockade; ISA—intrinsic sympathomimetic activity; Gen—generation.

A detailed classification of β-blockers and their pharmacological characteristics is presented in Figure 1.

Despite pharmacological differences, β-blockers primarily exert their therapeutic effects by reducing cardiac workload, lowering blood pressure, and stabilizing heart rhythm, making them a cornerstone of cardiovascular disease management [47,49,50]. However, accumulating evidence suggests that β-AR blockade may also exert biological effects beyond the cardiovascular system, particularly in oncology. Mechanistic studies indicate that inhibition of β-adrenergic signaling can modulate the TME, suppress angiogenesis, and reduce cancer cell proliferation, migration, invasion, and metastatic potential, processes that are highly relevant to CRC progression [15,52,53].

Although several β-blockers have been investigated for their potential anticancer activity, PrOH has received the greatest attention in CRC research and therefore serves as the principal reference compound throughout thisreviewConsequently, PKA enhances cell proliferation, survival, and adaptation to cellular stress, thereby promoting cancer progressions review. As a non-selective β_1_/β_2_-AR antagonist, PrOH effectively inhibits β_2_-adrenergic signaling, the receptor subtype most strongly implicated in CRC progression, angiogenesis, EMT, metastasis, and modulation of the TME. Nevertheless, evidence regarding other β-blockers, including cardioselective and mixed α_1_/β-adrenergic antagonists, is discussed where appropriate to provide a balanced overview of this therapeutic class.

As a non-selective β-AR antagonist, PrOH has been shown to exert antitumor effects that extend beyond its established cardiovascular indications. These effects have been attributed to its ability to modulate key proliferative signaling pathways, inhibit proangiogenic responses, and influence both immune and stromal components of the TME [54]. In addition, PrOH has demonstrated the potential to enhance antitumor immunity and increase tumor sensitivity to conventional therapies [47,54].

Although the clinical application of β-blockers in oncology requires further validation in prospective clinical trials, growing experimental and clinical evidence suggests that β-AR blockade may slow tumor progression and improve treatment outcomes. Consequently, β-blockers, particularly PrOH, have emerged as promising candidates for drug repurposing strategies in CRC and other solid malignancies [15,47,54].

### 3.2. The Role of β_2_-Adrenergic Signaling in the Regulation of Colorectal Cancer Proliferation and Progression

Signaling mediated by the β_2_-AR plays a pivotal role in CRC progression, serving as an important molecular link between chronic psychological stress and tumor development. Activation of β_2_-AR by catecholamines, particularly Epi and NE, initiates several intracellular signaling pathways, including the cAMP/PKA, PI3K/Akt/mTOR, and MAPK/ERK cascades, which collectively promote cancer-cell proliferation, inhibit apoptosis, and support metabolic reprogramming [55,56]. Furthermore, β_2_-adrenergic signaling contributes to TME remodeling by enhancing angiogenesis through increased VEGF expression and by promoting EMT, thereby facilitating tumor invasion and metastatic dissemination. Elevated β_2_-AR expression has been associated with advanced disease stage and unfavorable clinical outcomes, highlighting this pathway as a promising therapeutic target in CRC, particularly in the context of β-blocker-based interventions [54].

#### 3.2.1. β_2_-Adrenergic Signaling and the cAMP/PKA/CREB Pathway

Activation of β_2_-AR initiates a G protein-dependent signaling cascade at the cell membrane. Ligand binding stimulates adenylyl cyclase, resulting in an increase in intracellular cyclic adenosine monophosphate (cAMP), a key second messenger that activates protein kinase A (PKA), the principal effector of this pathway [47,55,56,57]. Activated PKA subsequently phosphorylates members of the cAMP response element-binding protein (CREB) and activating transcription factor (ATF) families, thereby inducing transcriptional programs associated with tumor growth, cellular adaptation to stress, and malignant progression [55,56,58]. Sustained β_2_-AR signaling promotes DNA damage accumulation, suppresses p53 activity, enhances angiogenesis, and increases resistance to programmed cell death [17,57]. Collectively, these alterations contribute to the development of an aggressive tumor phenotype characterized by uncontrolled proliferation, enhanced survival, and increased invasive potential. Although much of the mechanistic understanding of the β_2_-AR/cAMP/PKA signaling axis originates from studies in other cancer models, including glioblastoma and non-malignant epithelial cells such as THLE-2, accumulating evidence indicates that this pathway also plays a pivotal role in CRC.

In CRC, chronic activation of the β_2_-AR/cAMP/PKA signaling axis facilitates malignant cell growth and adaptation to adverse microenvironmental conditions. Moreover, deregulation of CREB- and ATF-dependent transcription further amplifies oncogenic signaling networks, thereby promoting disease progression, metastatic dissemination, and therapeutic resistance [17,55,56,57,58]. PKA-mediated phosphorylation generates the active form of CREB (p-CREB), which binds to cAMP response element (CRE) sequences within the promoters of numerous genes involved in CRC progression [59,60]. Among these are the oncogenes *CCND1* and *MYC*, which stimulate cancer cell proliferation, *B-cell lymphoma 2* (*BCL-2*), which enhances cell survival by inhibiting apoptosis, *VEGF*, a key regulator of angiogenesis, and the matrix metalloproteinases *MMP2* and *MMP9*, which promote extracellular matrix (ECM) degradation, facilitating tumor invasion and migration [61,62]. Collectively, activation of CREB initiates a transcriptional program that promotes sustained proliferation, resistance to apoptosis, angiogenesis, and increased metastatic potential of CRC cells. Experimental studies have further demonstrated that pharmacological inhibition of β_2_-ARs with PrOH suppresses this signaling pathway by reducing CREB phosphorylation and transcriptional activity, thereby decreasing the expression of CREB-regulated genes and ultimately limiting tumor growth, invasion, and metastatic potential [61,63].

**Figure 2:**
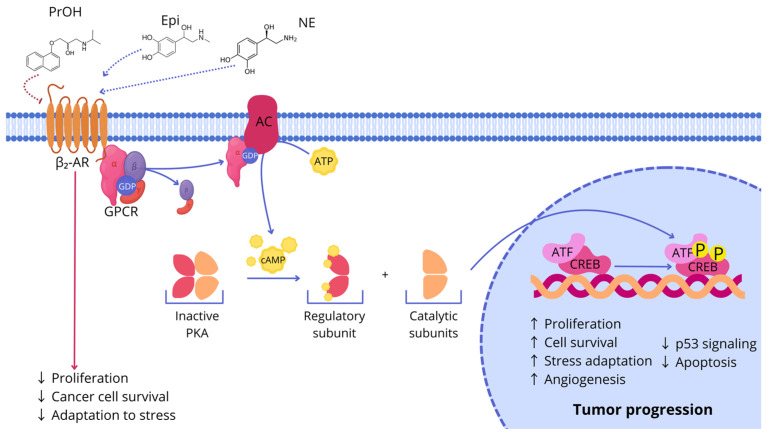
Schematic representation of β_2_-adrenergic receptor (β_2_-AR)-mediated activation of the cAMP/PKA/CREB signaling pathway and its inhibition by propranolol [53,56,57,58,64]. Blue solid arrows indicate intracellular signaling activation, whereas blue dashed arrows indicate ligand–receptor interactions. Red dashed arrows indicate receptor blockade by propranolol, and red solid arrows indicate the inhibitory biological effects of β-blockade. Yellow circles labeled “P” indicate phosphorylation. Symbols ↑ and ↓ denote increased and decreased expression or biological activity, respectively. **Abbreviations:** AC—adenylyl cyclase; ATP—adenosine triphosphate; ATF—activating transcription factor; β_2_-AR—beta-2 adrenergic receptor; cAMP—cyclic adenosine monophosphate; CREB—cAMP response element-binding protein; Epi—epinephrine (adrenaline); GPCR—G protein-coupled receptor; Gα—G protein alpha subunit; Gβ—G protein beta subunit; GDP—guanosine diphosphate; NE—norepinephrine (noradrenaline); p53—tumor protein p53; PKA—protein kinase A; PrOH—propranolol.

The detailed mechanism of β_2_-AR-mediated activation of the cAMP/PKA/CREB pathway and the inhibitory effects of β-blockers are illustrated in Figure 2.

#### 3.2.2. β_2_-Adrenergic Signaling and the PI3K/Akt Pathway

Although the β_2_-AR/cAMP/PKA pathway represents a central component of adrenergic signaling, its biological effects are not restricted to this signaling axis alone. Instead, β_2_-AR activation functionally interacts with several oncogenic pathways, including the phosphoinositide 3-kinase/protein kinase B (PI3K/Akt) cascade, thereby amplifying proliferative and pro-survival signaling [57,65,66]. This extensive signaling cross-talk is particularly relevant in endothelial cells and in CRC, where dysregulation of the PI3K/Akt pathway is a common event associated with tumor progression, angiogenesis, and therapeutic resistance [56,57,67].

Dysregulation of the PI3K/Akt pathway is a hallmark of CRC and frequently results from activating mutations in the *PIK3CA* gene or loss of function of the tumor suppressor phosphatase and tensin homolog (PTEN), a key negative regulator of this signaling cascade. Both molecular alterations lead to constitutive, ligand-independent activation of the PI3K/Akt/mTOR pathway, promoting autonomous cancer cell survival, proliferation, metabolic adaptation, and resistance to anticancer therapy [68,69].

Phosphoinositide 3-kinases (PI3Ks) comprise a family of lipid kinases that phosphorylate membrane phospholipids and regulate multiple intracellular signaling processes. Although PI3Ks are classified into several groups based on their structure and biological functions, class I PI3Ks are considered the most relevant in cancer biology [57,67,70]. These enzymes are activated by extracellular stimuli, including receptor tyrosine kinases (RTKs) and G-protein-coupled receptors, and function as heterodimeric complexes consisting of catalytic and regulatory subunits. Because of their central role in regulating cell growth, proliferation, metabolism, and survival, aberrant PI3K signaling is closely associated with cancer development and progression [65,67].

Activation of the PI3K/Akt pathway is frequently initiated by stimulation of RTKs such as EGFR. Following ligand binding, these receptors activate a multistep signaling cascade that culminates in Akt activation. As a key downstream effector, Akt promotes cell survival, proliferation, migration, and resistance to apoptosis. Moreover, the PI3K/Akt pathway regulates the transcriptional activity, stability, and expression of ARs, thereby contributing to reciprocal interactions between growth factor and adrenergic signaling networks. In CRC cells, excessive activation of PI3K/Akt signaling enhances proliferative capacity and promotes resistance to anticancer therapies [57,67,71].

Persistent activation of the PI3K/Akt pathway contributes to tumor progression through multiple mechanisms, including cyclin D1 dysregulation, impairment of p53-mediated tumor suppressor functions, and increased expression of matrix metalloproteinase-2 (MMP-2), which facilitates ECM degradation and promotes tumor invasion [67,71]. These findings provide a mechanistic rationale for targeting β_2_-adrenergic signaling as an adjunctive therapeutic strategy. Pharmacological inhibition of β_2_-AR may attenuate cAMP-dependent signaling and indirectly modulate PI3K/Akt pathway activity, thereby suppressing tumor growth, angiogenesis, and prosurvival signaling. Consequently, β-blockers, particularly PrOH, are being investigated as promising adjunctive agents capable of disrupting key signaling pathways involved in CRC progression and therapeutic resistance [55,56,57,58,67].

#### 3.2.3. β_2_-Adrenergic Signaling and the RAS/RAF/MEK/ERK Pathway

Another major signaling cascade involved in CRC progression is the RAS/RAF/MEK/ERK pathway, which plays a fundamental role in regulating cell proliferation, survival, differentiation, and maintenance of the malignant phenotype. Under physiological conditions, this pathway is primarily activated by growth factors acting through receptor tyrosine kinases, including EGFR. However, accumulating evidence suggests that β_2_-adrenergic signaling may also contribute to activation of this pathway, thereby linking chronic stress responses with oncogenic signaling in CRC [64,65,70].

Extracellular signal-regulated kinases 1 and 2 (ERK1/2) are serine/threonine kinases activated through phosphorylation by mitogen-activated protein kinases 1 and 2 (MEK1/2). Following activation, phosphorylated ERK (pERK) translocates to the nucleus, where it regulates the activity of multiple transcription factors, including ETS-like protein 1 (Elk-1), resulting in the expression of genes involved in cell-cycle progression, proliferation, and survival [64,70,71,72,73]. ERK signaling is particularly important for CRC progression because it promotes transition through the G1/S cell-cycle checkpoint and stimulates the synthesis of proteins required for DNA replication and cellular growth. Consequently, persistent ERK activation enhances proliferative capacity and increases resistance to apoptotic stimuli [64].

In CRC, activation of the RAS/RAF/MEK/ERK pathway is frequently driven by mutations in upstream oncogenes, particularly members of the RAS gene family. Under normal physiological conditions, Ras proteins cycle between an inactive guanosine diphosphate (GDP)-bound state and an active guanosine triphosphate (GTP)-bound state in response to extracellular signals. However, oncogenic *RAS* mutations result in constitutive activation of Ras proteins, rendering signaling independent of external growth stimuli and leading to persistent activation of downstream ERK signaling [64,70,74]. Such alterations occur in a substantial proportion of CRC cases and represent key molecular drivers of tumor progression [18].

Importantly, β_2_-adrenergic signaling may functionally interact with the constitutively activated RAS/RAF/MEK/ERK pathway, further amplifying proliferative and pro-survival signaling. Through this mechanism, chronic adrenergic stimulation may contribute to the development of a more aggressive tumor phenotype characterized by enhanced growth, invasion, and metastatic potential. Consequently, β-blockers may attenuate hormonally induced ERK activation and reduce downstream oncogenic signaling. Given the central role of the RAS/RAF/MEK/ERK pathway in CRC pathogenesis, inhibition of β-adrenergic signaling represents a potentially valuable complementary strategy for limiting tumor progression and improving therapeutic responsiveness [64,70,74].

#### 3.2.4. Cross-Talk Between PI3K/Akt and RAS/RAF/MEK/ERK Signaling

The functional interdependence and synergistic interaction between the RAS/RAF/MEK/ERK and PI3K/Akt/mTOR signaling pathways constitute a fundamental mechanism of oncogenic signal transduction in CRC. These interconnected signaling networks are characterized by numerous feedback loops and cross-regulatory interactions, which enable cancer cells to maintain proliferative and survival signaling despite inhibition of individual pathway components. Consequently, this molecular cross-talk contributes to multidrug resistance, reduced sensitivity to physiological antiproliferative signals, impaired apoptotic responses, and ultimately uncontrolled tumor growth [57,59,64].

**Figure 3:**
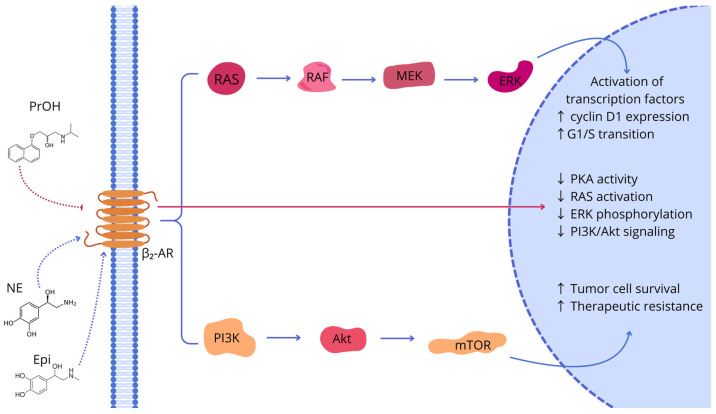
Schematic representation of β_2_-adrenergic receptor (β_2_-AR)-mediated regulation of the PI3K/Akt/mTOR and RAS/RAF/MEK/ERK signaling pathways and their modulation by β-blockers [49,57,70,71,72,73,74]. Blue solid arrows indicate intracellular signaling activation, whereas blue dashed arrows indicate ligand–receptor interactions. Red dashed arrows indicate receptor blockade by propranolol, and red solid arrows indicate the inhibitory biological effects of β-blockade. Symbols ↑ and ↓ denote increased and decreased expression or biological activity, respectively. **Abbreviations:** Akt—protein kinase B; β_2_-AR—beta-2 adrenergic receptor; Epi—epinephrine (adrenaline); ERK—extracellular signal-regulated kinase; G1/S transition—cell-cycle transition from G1 to S phase; MEK—mitogen-activated protein kinase kinase; mTOR—mechanistic target of rapamycin; NE—norepinephrine (noradrenaline); PI3K—phosphoinositide 3-kinase; PKA—protein kinase A; PrOH—propranolol; RAF—proto-oncogene serine/threonine-protein kinase; RAS—rat sarcoma virus GTPase.

A detailed model of the interactions and points of convergence between these pathways is presented in Figure 3.

One important regulator of this signaling network is PKA, which is activated downstream of β_2_-AR signaling. PKA modulates both the MAPK and PI3K/Akt pathways through phosphorylation of selected Raf kinase isoforms, regulation of Src family kinases, and modulation of RTK-associated adaptor proteins. Consequently, PKA enhances cell proliferation, survival, and adaptation to cellular stress, thereby promoting cancer progression Furthermore, PKA may indirectly stimulate PI3K/Akt signaling through RTK-dependent mechanisms and modulation of intracellular regulatory proteins. Activation of the PI3K/Akt pathway subsequently promotes protein synthesis, cellular metabolism, and mTOR-dependent survival signaling, collectively supporting tumor growth and resistance to therapy [57,59,64].

The interaction between β_2_-adrenergic signaling and the MAPK pathway is particularly relevant in *KRAS*-mutant CRC. In these tumors, mutant KRAS remains constitutively active in its GTP-bound state, resulting in persistent activation of the RAF/MEK/ERK cascade independently of receptor tyrosine kinases such as EGFR. Nevertheless, chronic β_2_-AR stimulation provides an additional layer of stress-induced MAPK activation. Mechanistically, activation of the cAMP/PKA and Src signaling cascades promotes c-Raf phosphorylation, thereby facilitating further activation of MEK1/2 and ERK1/2 independently of upstream RTK signaling [75,76]. Consequently, β_2_-adrenergic signaling may further potentiate oncogenic signaling even in the presence of constitutively active *KRAS*.

In *KRAS*-mutant CRC, which is intrinsically resistant to EGFR-targeted therapies, non-selective β-blockers such as PrOH may attenuate adrenergic amplification of oncogenic signaling by suppressing the cAMP/PKA and Src pathways. This may reduce excessive ERK activation while simultaneously limiting activation of additional signaling networks, including PI3K/Akt, thereby decreasing stress-induced chemoresistance, metabolic adaptation, and metastatic potential [77].

Therapeutic management of *KRAS*-mutant CRC is further complicated by the emergence of adaptive resistance to direct KRAS inhibitors, including KRAS^G12C^ inhibitors. Pharmacological inhibition of mutant *KRAS* frequently results in rapid compensatory reactivation of the MAPK pathway through EGFR-dependent feedback activation and signaling mediated by wild-type RAS isoforms, a phenomenon referred to as MAPK rebound [78]. Simultaneously, cancer cells may activate alternative RAS-independent survival pathways, including the RHO-ROCK cascade, which promotes activation of the YAP/TEAD transcriptional program [79]. Because adrenergic signaling through the cAMP/PKA/Src axis overlaps with these adaptive mechanisms and contributes to cytoskeletal remodeling, combining β-blockers with KRAS-targeted therapies has been proposed as a potential strategy for overcoming therapeutic resistance. Pharmacological suppression of adrenergic signaling may attenuate EGFR-dependent feedback activation and limit RHO-ROCK/YAP signaling, thereby potentially restoring sensitivity to KRAS-targeted therapies. However, this concept remains investigational and requires further validation in preclinical and clinical studies.

These observations suggest that the therapeutic effects of β-blockers may differ according to KRAS mutation status, primarily because they target distinct signaling mechanisms rather than the constitutively active KRAS protein itself. Whereas in *KRAS*-wild-type tumors β-blockers may enhance the efficacy of anti-EGFR therapies by suppressing stress-induced EGFR transactivation, in *KRAS*-mutant CRC their potential therapeutic benefit is more likely associated with attenuation of adrenergic amplification of parallel pro-survival signaling pathways, including the cAMP/PKA/Src and PI3K/Akt axes. However, current evidence supporting these differential mechanisms is derived predominantly from preclinical studies, and prospective clinical investigations are needed to determine whether *KRAS* mutation status represents a predictive biomarker of response to β-blocker therapy.

Importantly, in KRAS-wild-type tumors, MAPK activation remains largely dependent on membrane receptor signaling, including stress-induced EGFR transactivation. Under these conditions, β-blockers may inhibit adrenergic stimulation of EGFR and enhance the therapeutic efficacy of anti-EGFR monoclonal antibodies, such as cetuximab and panitumumab [77,80,81].

Given the central role of β_2_-adrenergic signaling in regulating these pathways, pharmacological inhibition of β-ARs represents a promising strategy for limiting oncogenic signal transduction. Pharmacological blockade of β-ARs by β-blockers, particularly PrOH, may reduce intracellular cAMP levels and attenuate PKA activity, thereby suppressing proliferative and pro-survival signaling mediated through both the RAS/RAF/MEK/ERK and PI3K/Akt/mTOR pathways. Experimental studies further suggest that β-blockers may restore sensitivity to apoptotic stimuli while simultaneously suppressing angiogenesis, invasion, and metastatic dissemination in CRC. Nevertheless, the precise molecular mechanisms underlying these effects remain incompletely understood and require further validation in preclinical and clinical studies [53,57,59,64].

#### 3.2.5. β-Arrestin-Mediated Signaling and Receptor Desensitization

β-Arrestins are multifunctional intracellular adaptor proteins that play a central role in regulating GPCR signaling, including β_2_-AR signaling. Beyond their classical role in receptor desensitization, β-arrestins function as signaling scaffolds that coordinate multiple intracellular pathways involved in cell proliferation, survival, migration, and differentiation. Two ubiquitously expressed isoforms have been identified, β-arrestin-1 and β-arrestin-2. Their activity is tightly regulated by post-translational modifications. Under basal conditions, both isoforms are constitutively phosphorylated, whereas agonist-induced dephosphorylation regulates receptor desensitization, endocytosis, and recycling. Furthermore, ubiquitination of β-arrestins stabilizes the β-arrestin–GPCR complex, thereby prolonging β-arrestin-dependent signaling, particularly through MAPK pathways [82,83,84].

Tight regulation of GPCR signaling is essential for maintaining cellular homeostasis, whereas excessive or prolonged receptor activation may promote aberrant proliferation, survival, and malignant transformation. To prevent these pathological consequences, evolutionarily conserved desensitization mechanisms attenuate cellular responses during continuous or repeated ligand stimulation. GPCR desensitization comprises both rapid and long-term regulatory processes. Short-term desensitization, occurring within minutes, results from β-arrestin-mediated steric inhibition of receptor–G protein coupling. In contrast, long-term desensitization, developing over hours to days, involves receptor internalization, lysosomal degradation, and reduced receptor gene expression. Both processes are initiated by G protein-coupled receptor kinases (GRKs), which phosphorylate agonist-activated GPCRs at their C-terminal tails or intracellular loop regions, generating high-affinity docking sites for β-arrestins. Because β-arrestins bind to receptor domains that overlap with G protein interaction sites, their recruitment rapidly terminates canonical G protein-dependent signaling [82,85,86].

In addition to uncoupling GPCRs from G proteins, β-arrestins actively regulate receptor signaling by recruiting enzymes responsible for the local degradation of second messengers. Upon β_2_-AR activation, β-arrestins recruit cyclic nucleotide phosphodiesterases (PDEs), which rapidly hydrolyze cAMP to AMP, thereby attenuating PKA signaling and preventing PKA-dependent feedback phosphorylation of the receptor. PDE recruitment exhibits marked isoform specificity; for example, PDE4D8 constitutively associates with β_1_-AR independently of β-arrestins and dissociates following agonist stimulation, whereas recruitment to β_2_-AR is β-arrestin dependent. In parallel, β-arrestins recruit diacylglycerol kinases that convert diacylglycerol into phosphatidic acid, thereby terminating protein kinase C (PKC) signaling while simultaneously initiating alternative downstream signaling events. Consequently, β-arrestin-mediated desensitization represents an active signaling process that not only terminates G protein-dependent signaling but also initiates distinct G protein-independent signaling pathways [87].

Beyond receptor regulation, β-arrestins function as multifunctional signaling scaffolds that modulate numerous processes involved in cancer progression. They regulate cell proliferation, promote migration and invasion, enhance cell survival through anti-apoptotic signaling, and contribute to angiogenesis, drug resistance, EMT, and metastatic dissemination. Experimental silencing of β-arrestins consistently suppresses EMT, reduces matrix metalloproteinase (MMP2 and MMP9) activity, and diminishes the metastatic potential of cancer cells. Collectively, these findings identify β-arrestin-mediated signaling as a promising therapeutic target in oncology [88].

In CRC, β_2_-AR activation promotes tumor progression through two complementary signaling axes: the canonical cAMP/PKA/CREB pathway and β-arrestin-dependent signaling, including MAPK and PI3K/AKT cascades. This signaling complexity provides additional mechanistic support for targeting β-adrenergic signaling in CRC. Consequently, balanced (unbiased) β-blockers, such as PrOH, may simultaneously inhibit both G protein-dependent and β-arrestin-mediated signaling. Alternatively, selective modulation of β-arrestin-dependent signaling may represent a complementary therapeutic strategy for more effectively disrupting adrenergic signaling networks that drive CRC progression.

### 3.3. Effects on the Tumor Microenvironment, Immune Regulation and Angiogenesis

One of the principal mechanisms through which adrenergic signaling contributes to CRC progression is the activation of cancer-associated fibroblasts (CAFs), leading to stromal remodeling and the establishment of a tumor-supportive microenvironment. β-Adrenergic stimulation enhances the secretion of pro-inflammatory and pro-angiogenic mediators, including interleukin-6 (IL-6), VEGF, and MMPs, thereby promoting ECM remodeling, tumor invasion, and metastatic dissemination [89,90]. In CRC, increased stromal activation and desmoplasia are strongly associated with poor prognosis, enhanced metastatic potential, and resistance to anticancer therapy. Consequently, β-blockers may exert antitumor effects not only through direct inhibition of cancer-cell signaling but also by disrupting the stromal niche that supports tumor growth, invasion, and disease progression [15].

Among the various effects of β-blockers on the TME, inhibition of angiogenesis has attracted considerable attention. Angiogenesis is a hallmark of CRC progression, providing the oxygen and nutrients necessary for sustained tumor growth and metastatic dissemination. β-Adrenergic signaling promotes angiogenesis primarily through activation of the cAMP/PKA signaling cascade, leading to increased expression of VEGF, hypoxia-inducible factor-1α (HIF-1α), and other pro-angiogenic mediators [15,91]. Consequently, β-adrenergic stimulation enhances endothelial-cell proliferation, migration, and vascular permeability, whereas pharmacological β-blockade suppresses endothelial activation, reduces microvessel density, and limits tumor neovascularization. Among the available β-blockers, PrOH has demonstrated particularly strong anti-angiogenic activity by inhibiting angiogenic signaling and potentiating the effects of conventional anticancer therapies [92]. In vivo studies further showed that PrOH reduced VEGFA expression by up to 50%, providing additional evidence of its anti-angiogenic potential [93]. However, the magnitude of these effects appears to depend on several tumor-specific factors, including the degree of hypoxia, inflammatory activity, β-AR expression patterns, and tumor stage.

β-Blockers can also modulate the immunological landscape of the TME. Experimental studies indicate that PrOH enhances systemic antitumor immunity by promoting the recruitment and activation of tumor-infiltrating lymphocytes. In vivo administration of PrOH has been associated with increased CD4^+^ T-cell infiltration, enhanced interferon-γ (IFN-γ) production, and a reduction in immunosuppressive regulatory T cells (Tregs), collectively contributing to a more immunostimulatory TME [74]. Furthermore, PrOH treatment has been reported to reduce splenic PD-1 expression, suggesting partial reversal of T-cell exhaustion [74].

Similarly, other experimental studies have demonstrated enhanced infiltration of CD4^+^ T cells together with a trend toward increased CD8^+^ T-cell recruitment following PrOH treatment. Although the proportion of Tregs relative to CD4^+^ T cells remained unchanged, increased numbers of activated CD137^+^PD-1^+^ CD4^+^ T cells and a trend toward higher PD-1^+^ CD8^+^ T-cell populations were observed, indicating enhanced immune activation despite persistent features of T-cell exhaustion [93].

Emerging evidence further suggests that β-adrenergic signaling may directly regulate immune checkpoint expression within the TME. Chronic activation of β_2_-ARs by catecholamines has been associated with increased expression of PD-L1 on both tumor and immune cells through activation of the cAMP/PKA, STAT3, and NF-κB signaling pathways. Elevated PD-L1 expression promotes T-cell exhaustion, suppresses cytotoxic CD8^+^ T-cell activity, and facilitates immune evasion. Consequently, pharmacological β-adrenergic blockade, particularly with non-selective β-blockers such as PrOH, may attenuate adrenergic-induced PD-L1 expression, enhance antitumor immune responses, and potentially improve the efficacy of immune checkpoint inhibitors. Collectively, these findings provide a strong rationale for exploring combination strategies involving β-blockers and immune checkpoint inhibitors, including pembrolizumab and nivolumab, in CRC [94]. However, current evidence is derived predominantly from preclinical studies, and further clinical investigations are required to determine the therapeutic significance of this therapeutic approach.

Hypoxia represents a hallmark feature of the CRC TME and is closely associated with angiogenesis, metabolic adaptation, and treatment resistance. Under hypoxic conditions, stabilization of hypoxia-inducible factor-1α (HIF-1α) promotes the transcription of VEGF and numerous genes involved in metabolic adaptation, cell survival, and neovascularization [95]. Emerging evidence indicates that adrenergic signaling may further amplify hypoxia-associated pathways, thereby exacerbating angiogenic responses and promoting the formation of abnormal, highly permeable tumor vasculature [96]. Consequently, β-blockers may partially counteract these effects by attenuating adrenergic activation of the HIF-1α/VEGF signaling axis, thereby limiting hypoxia-driven angiogenesis and tumor progression.

Another important component of vascular remodeling and normalization is the angiopoietin/Tie2 signaling axis, which plays a central role in regulating vascular stability and maturation. Angiopoietin-1 (Ang-1) promotes vessel maturation and stabilization, whereas angiopoietin-2 (Ang-2) destabilizes the vasculature, thereby facilitating angiogenic remodeling. A balanced interplay between Ang-1 and Ang-2 is therefore essential for maintaining vascular homeostasis [97].

The angiopoietin/Tie2 pathway closely interacts with platelet-derived growth factor (PDGF) signaling, which contributes to tumor vascular remodeling by promoting pericyte recruitment through PDGFRβ-dependent mechanisms, thereby influencing the stability and functionality of tumor blood vessels [98]. Moreover, simultaneous targeting of the PDGF/PDGFR and VEGF signaling pathways has been shown to enhance vascular normalization, reduce vessel leakiness, and improve tumor perfusion and oxygenation, highlighting the therapeutic potential of targeting multiple pro-angiogenic pathways concurrently [99].

In addition, β_2_-adrenergic signaling has been implicated in the regulation of EMT, suggesting that adrenergic activation contributes not only to vascular remodeling but also to tumor cell plasticity, invasion, and metastatic progression [98,99,100].

These observations have led to the hypothesis that β-blockade may contribute to vascular normalization, a process characterized by improved vessel maturity, reduced vascular permeability, and enhanced tissue perfusion. Such changes may facilitate the delivery of chemotherapeutic agents while reducing hypoxia-driven mechanisms of therapeutic resistance. This concept is particularly relevant in the context of anti-angiogenic therapies targeting the VEGF signaling pathway and provides a strong rationale for combining β-blockers with established anti-angiogenic treatment strategies in CRC. Both preclinical and clinical studies have suggested that combining PrOH with tyrosine kinase inhibitors may improve treatment response and overall survival (OS) [62,92,101].

**Figure 4:**
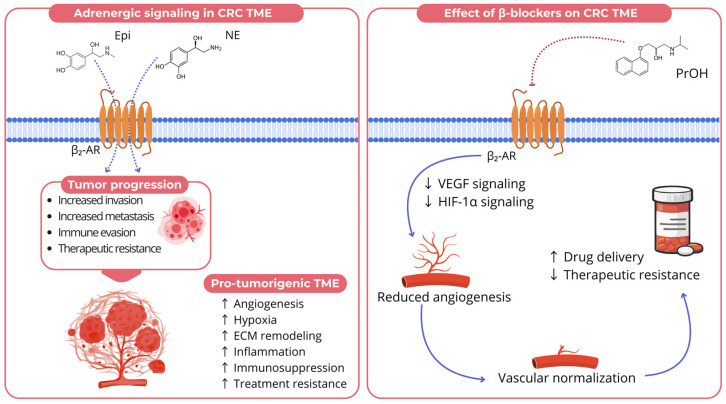
Proposed effects of β-blockers on the colorectal cancer tumor microenvironment (TME), illustrating attenuation of adrenergic signaling, inhibition of angiogenesis and hypoxia-associated pathways, modulation of stromal remodeling, vascular normalization, and reduced therapeutic resistance [15,89,90,95]. Blue dashed arrows indicate ligand–receptor interactions; red dashed arrows indicate receptor blockade by propranolol; blue solid arrows indicate the downstream biological effects of β-blockade. Symbols ↑ and ↓ denote increased and decreased expression or biological activity, respectively. **Abbreviations:** β_2_-AR—beta-2 adrenergic receptor; CRC—colorectal cancer; ECM—extracellular matrix; Epi—epinephrine; HIF-1α—hypoxia-inducible factor 1-alpha; NE—norepinephrine; PrOH—propranolol; TME—tumor microenvironment; VEGF—vascular endothelial growth factor.

Despite these promising findings, the clinical significance of β-blockers in oncology remains to be fully established. Although several observational studies have reported improved survival, particularly among patients with advanced-stage CRC, other analyses have not demonstrated significant effects on postoperative outcomes or CRC incidence [102,103,104]. These inconsistencies may reflect differences in study design, patient populations, tumor stage, concomitant therapies, and the presence of shared risk factors for both cardiovascular disease and cancer progression [105,106,107]. Therefore, adequately powered prospective randomized clinical trials are required to determine whether β-blockers, particularly PrOH, can provide clinically meaningful benefits as components of multimodal treatment strategies for CRC. The proposed mechanisms through which β-blockers modulate the CRC TME are summarized in Figure 4.

### 3.4. Modulation of Epithelial–Mesenchymal Transition and Metastasis

Cancer metastasis is a complex, multistep process that depends on the intrinsic properties of tumor cells, the TME, and systemic regulatory signals. In CRC, EMT is recognized as a key mechanism driving tumor progression and metastatic dissemination. EMT is a dynamic cellular reprogramming process that plays essential roles in embryonic development and tissue repair under physiological conditions. However, its aberrant activation in cancer promotes the acquisition of invasive and migratory properties, facilitating tumor spread [108,109].

The phenotypic plasticity associated with EMT enables CRC cells to adapt to the changing conditions encountered during the metastatic cascade [110]. A hallmark of EMT is the increased expression and activity of MMPs, which degrade EMC components and facilitate cancer-cell invasion and migration [108,109,110,111]. Consequently, inhibition of EMT and MMP activity has emerged as an attractive therapeutic strategy for limiting metastatic progression.

Accumulating evidence indicates that the neurohormonal stress response is an important regulator of EMT in CRC. Chronic exposure to stress-associated catecholamines and sustained activation of β-adrenergic signaling have been shown to promote EMT, thereby enhancing the migratory and invasive potential of CRC cells [111]. Recent evidence suggests that the pro-tumorigenic effects of adrenergic signaling may be mediated, at least in part, through TRIM2-dependent activation of NF-κB signaling. In CRC models, Epi was shown to upregulate TRIM2 expression, resulting in IκBα ubiquitination and degradation, followed by activation of the NF-κB pathway. These molecular events promoted tumor growth and M2 polarization of tumor-associated macrophages (TAMs), thereby contributing to a tumor-promoting microenvironment and CRC progression [112,113].

Growing evidence also suggests that neural remodeling within the TME contributes to CRC progression by reinforcing the bidirectional crosstalk between the nervous system and tumor cells. In a co-culture model of dorsal root ganglia and mCRC cells, neuronal projections were markedly increased compared with dorsal root ganglia cultured alone, and tumor cells exhibited directional migration along these neuronal projections [114]. Consistent with these experimental findings, analyses of patient tumor specimens demonstrated that increased intratumoral nerve infiltration was associated with poorer OS and disease-free survival (DFS), suggesting that tumor-associated neurogenesis may serve as a marker of aggressive disease and unfavorable clinical outcomes in CRC [114].

β-Adrenergic signaling also interacts with transforming growth factor-beta (TGF-β), one of the most potent inducers of EMT. Activation of β-AR promotes increased TGF-β secretion by both tumor cells and components of the TME, leading to activation of Smad-dependent signaling pathways and repression of E-cadherin expression [57,110]. The combined activity of these mechanisms enhances cellular plasticity, stabilizes the mesenchymal phenotype, and promotes metastatic dissemination [110].

Pharmacological inhibition of β-adrenergic signaling by β-blockers has been shown to attenuate stress-induced EMT and reduce the invasive behavior of CRC cells. β-Blockers suppress activation of the β-adrenergic/TGF-β axis, decrease MMP activity, and limit ECM degradation, thereby reducing cancer-cell migration and metastatic potential [57,110]. Through their ability to modulate key pathways involved in EMT and metastasis, β-blockers may represent promising adjunctive agents capable of limiting neurohormonal-dependent CRC progression and dissemination [108,110,111,112].

### 3.5. Induction of Apoptosis and Autophagy

Apoptosis, or programmed cell death, is a fundamental biological process responsible for maintaining tissue homeostasis through the elimination of damaged, dysfunctional, or potentially malignant cells. Dysregulation of apoptotic pathways is a hallmark of cancer and contributes significantly to CRC development, progression, and treatment resistance [115,116]. Apoptosis is primarily mediated through two major signaling pathways: the extrinsic (death receptor-mediated) pathway and the intrinsic (mitochondrial) pathway. The latter is activated by intracellular stress signals and is tightly regulated by members of the BCL-2 protein family, which control mitochondrial outer membrane permeability [115,116].

The BCL-2 family comprises three functionally distinct groups of proteins. The first group consists of anti-apoptotic proteins, including BCL-2, BCL-XL, and MCL-1, which preserve mitochondrial integrity and prevent cytochrome c release. Among these, BCL-2 appears to play a particularly important role in CRC, where its overexpression promotes cancer-cell survival and contributes to resistance to chemotherapy and radiotherapy [116,117]. The second group includes pro-apoptotic effector proteins such as BAX and BAK, which induce mitochondrial membrane permeabilization following activation. However, in CRC cells, their activity is frequently suppressed because of the predominance of anti-apoptotic signaling pathways [116,117,118,119].

The third group comprises BH3-only proteins, which act as critical regulators of the balance between pro- and anti-apoptotic members of the BCL-2 family. Disturbance of this balance, particularly through overexpression of anti-apoptotic proteins, impairs the physiological elimination of genetically damaged cells. Consequently, apoptosis resistance promotes mutation accumulation, tumor progression, metastatic dissemination, and reduced sensitivity to anticancer therapy [118].

Accumulating evidence indicates that β-adrenergic signaling contributes to apoptosis resistance through activation of the cAMP/PKA pathway and downstream oncogenic signaling networks, including PI3K/AKT and RAS/RAF/MEK/ERK. These pathways promote tumor-cell survival by enhancing the expression of anti-apoptotic proteins while suppressing pro-apoptotic signaling [55,57,67,70]. As a result, cancer cells exhibit increased resistance to cellular stress, chemotherapy, and apoptosis-inducing stimulation [118,119].

Pharmacological inhibition of β-ARs by β-blockers, particularly non-selective agents such as PrOH, suppresses cAMP/PKA signaling and attenuates survival pathways including AKT and MAPK. This leads to a shift in the balance of BCL-2 family proteins toward a pro-apoptotic phenotype, characterized by increased BAX expression and reduced BCL-2 expression. Consequently, mitochondrial outer membrane permeabilization is promoted, resulting in cytochrome c release and activation of the intrinsic apoptotic pathway [14,118,119].

Experimental studies have demonstrated a direct association between β-blocker treatment and regulation of BCL-2 family proteins. In particular, PrOH has been shown to significantly reduce BCL-2 expression while increasing BAX expression and caspase-3 activation, providing molecular evidence of apoptosis induction through the mitochondrial pathway [120,121]. Similar observations have been reported in several cancer models, where PrOH-mediated inhibition of AKT signaling reduced anti-apoptotic activity and enhanced cellular susceptibility to apoptotic death [14,120,122]. These findings suggest that β-blockers may serve as promising adjuvant agents, particularly in tumors characterized by overexpression of anti-apoptotic BCL-2 family proteins.

Beyond classical apoptosis and autophagy, increasing evidence indicates that β-blockers may also modulate other forms of regulated cell death, including pyroptosis, ferroptosis, and necroptosis. Although these mechanisms remain incompletely understood, they may represent additional pathways contributing to the antitumor activity of β-blockers in CRC.

Emerging evidence suggests that apoptosis can be transformed into pyroptosis through caspase-3-mediated cleavage of gasdermin E (GSDME), a mechanism observed in response to several chemotherapeutic agents. Given that β-blockers inhibit pro-survival AKT/MAPK signaling while promoting intrinsic apoptosis through BAX upregulation and caspase-3 activation, they may also facilitate this apoptosis-to-pyroptosis transition in GSDME-expressing CRC cells. Subsequent GSDME cleavage results in plasma membrane pore formation, converting immunologically silent apoptosis into inflammatory pyroptosis and triggering the release of damage-associated molecular patterns (DAMPs) and pro-inflammatory cytokines, thereby potentially enhancing tumor immunogenicity [123].

Similarly, recent findings highlight a novel role for β-blockers in modulating ferroptosis in CRC. PrOH has been shown to induce canonical ferroptosis by disrupting iron homeostasis and lipid metabolism, leading to increased reactive oxygen species (ROS) accumulation and lipid peroxidation. Moreover, combination treatment with PrOH and capecitabine enhanced ferroptotic cell death and improved antitumor efficacy in preclinical CRC models [124]. In parallel, CD8^+^ T cell-derived IFN-γ promotes ferroptosis by suppressing the cystine/glutamate antiporter system. By enhancing antitumor immunity and remodeling the TME, β-blockers may indirectly increase the susceptibility of CRC cells to ferroptosis, thereby potentially improving responses to immunotherapy [125].

Although the relationship between β-adrenergic blockade and necroptosis has not been directly investigated in CRC, studies in other malignancies suggest a potential link. In prostate cancer models, β_2_-AR inhibition increased the expression of the necroptotic mediators receptor-interacting protein kinase 1 (RIPK1), receptor-interacting protein kinase 3 (RIPK3), and mixed lineage kinase domain-like protein (MLKL), thereby promoting necroptotic cell death. Given the immunogenic nature of necroptosis and its ability to stimulate antigen presentation and CD8^+^ T-cell activation through the release of DAMPs, β-blockade may represent a promising strategy that combines suppression of adrenergic signaling with induction of immunogenic tumor cell death. Nevertheless, direct evidence supporting this mechanism in CRC remains limited, and further mechanistic and clinical studies are required to clarify the role of β-blockers in regulating necroptosis in CRC [126].

### 3.6. Interactions with the Immune System

Recent advances in cancer neuroimmunology have identified the sympathetic nervous system as an important regulator of the TME. Chronic adrenergic stress profoundly influences antitumor immunity by reshaping cellular interactions within the TME and promoting the establishment of an immunosuppressive niche that favors tumor growth and progression [127]. Catecholamines, acting primarily through β_2_-ARs, mediate bidirectional communication between the nervous and immune systems. Because β_2_-ARs are expressed on numerous immune-cell populations, including T lymphocytes, natural killer (NK) cells, dendritic cells (DCs), macrophages, and myeloid-derived suppressor cells (MDSCs), adrenergic signaling can directly modulate immune-cell function within the TME [127,128,129].

One of the principal mechanisms through which adrenergic stress promotes immune evasion is the recruitment and activation of MDSCs. These immunosuppressive cells inhibit T-cell responses and facilitate tumor progression. Activation of β_2_-AR signaling enhances both the expansion and suppressive activity of MDSCs, partly through increased expression of arginase-1, which depletes extracellular arginine, an amino acid essential for effective T-cell activation and proliferation [127,130].

Adrenergic signaling also suppresses antitumor immunity through modulation of cytokine production. Activation of the β_2_-AR/cAMP/PKA signaling pathway promotes the secretion of anti-inflammatory mediators, particularly interleukin-10 (IL-10) and TGF-β. These cytokines inhibit T-cell proliferation and effector function while promoting the differentiation of CD4^+^ T cells into Tregs and T helper 2 (Th2) cells [100]. In parallel, adrenergic signaling drives macrophage polarization toward the M2 phenotype, generating TAMs that further reinforce the immunosuppressive microenvironment [131].

The accumulation of TAMs has profound consequences for antitumor immunity. These cells suppress the activity of cytotoxic CD8^+^ T lymphocytes and NK cells, impair dendritic-cell migration and antigen presentation, and contribute to the upregulation of immune checkpoint molecules such as PD-1 and CTLA-4 [104]. Moreover, TAMs secrete arginase-1 and indoleamine 2,3-dioxygenase (IDO), which deplete arginine and tryptophan, respectively. This metabolic deprivation promotes T-cell exhaustion, impairs effector-cell survival, and contributes to the development of profound immune dysfunction within the TME [127,132].

Upregulation of immune checkpoint signaling, particularly the PD-1/PD-L1 axis, represents an additional mechanism through which TAMs and MDSCs suppress antitumor immunity. Interaction of PD-L1 with PD-1 receptors expressed on T cells and NK cells induces functional exhaustion and impairs cytotoxic activity. Importantly, adrenergic signaling further enhances this process through activation of the β-AR/STAT3 pathway, which promotes PD-L1 stabilization and increased expression on myeloid cells, thereby reinforcing the immunosuppressive barrier within the TME [130,133,134,135]. Conversely, β-adrenergic blockade has been shown to reduce PD-L1 expression and restore T-cell-mediated antitumor immunity in several preclinical cancer models, providing an additional rationale for combining β-blockers with immune checkpoint inhibitors [136,137,138].

Emerging evidence indicates that inhibition of β-adrenergic signaling can partially reverse these immunosuppressive effects and promote the conversion of the TME into a more immunologically active state. This transition is characterized by increased infiltration of effector CD8^+^ T cells, reduced PD-1 expression, and a higher ratio of IFN-γ^+^ CD8^+^ T cells to Tregs. Such changes have been associated with enhanced antitumor immune responses and improved efficacy of immune checkpoint inhibitors, including anti-PD-1 therapies [93,136,137,138].

Collectively, these findings indicate that β_2_-adrenergic signaling plays a central role in shaping the immunosuppressive CRC microenvironment. Through regulation of MDSCs, TAMs, immune checkpoint expression, cytokine production, and macrophage polarization, adrenergic signaling facilitates tumor immune evasion and limits the effectiveness of antitumor immunity. Consequently, pharmacological inhibition of β-ARs by β-blockers may enhance immune surveillance, restore T-cell function, and increase tumor responsiveness to immunotherapeutic interventions.

Beyond the principal immunotherapeutic approaches investigated to date, ICIs have emerged as a major therapeutic advance in oncology. However, their efficacy in CRC remains largely restricted to MSI-H/dMMR tumors. In contrast, the vast majority of CRC cases are microsatellite-stable (MSS) and remain largely refractory to ICI monotherapy because of an immunologically “cold” TME characterized by limited CD8^+^ T-cell infiltration, accumulation of immunosuppressive myeloid cells and M2-like TAMs, together with PD-L1-mediated immune evasion. Consequently, considerable efforts have focused on developing combination strategies capable of overcoming these barriers and enhancing antitumor immune responses in MSS CRC [17].

Chronic psychophysical stress and sustained β-adrenergic signaling further exacerbate this immunosuppressive milieu. Therefore, pharmacological inhibition of β-ARs by β-blockers may represent a promising strategy to remodel the TME and enhance tumor responsiveness to immunotherapeutic interventions. By counteracting adrenergic signaling, β-blockers may enhance immune surveillance, restore CD8^+^ T-cell effector functions, reduce immunosuppressive cell populations, promote macrophage repolarization toward the M1 phenotype, and attenuate PD-L1-mediated immune evasion. Collectively, these mechanisms may convert immunologically “cold” MSS tumors into a more inflamed phenotype, thereby increasing their susceptibility to immune checkpoint blockade, although this concept remains to be validated in prospective preclinical and clinical studies [17,139].

This concept aligns closely with current efforts to sensitize MSS CRC to ICIs through combination strategies aimed at overcoming TME-mediated resistance. While clinical evaluation of β-blockers in this setting is ongoing, recent phase III evidence from the STELLAR-303 trial demonstrated that combining immunotherapy with a TME-modulating targeted agent may improve outcomes in patients with non-MSI-H/dMMR mCRC. These findings support the broader concept that successful immunotherapy combinations should simultaneously target immune checkpoint pathways and the immunosuppressive TME. In this context, β-blockers represent promising adjunctive agents capable of enhancing antitumor immunity and potentially improving the efficacy of ICIs, particularly in MSS CRC. Nevertheless, prospective mechanistic and clinical studies are required to validate this therapeutic strategy [139,140].

### 3.7. Synergy with Conventional Chemotherapy and Radiotherapy

Resistance to anticancer therapy remains one of the major challenges in CRC treatment and contributes substantially to disease progression and mortality [91]. Chronic psychophysical stress associated with cancer promotes sustained catecholamine release and persistent activation of adrenergic signaling pathways. Through β-AR activation, catecholamines stimulate angiogenesis, inflammation, tumor-cell survival, and metastatic dissemination, thereby creating conditions that diminish the effectiveness of conventional anticancer therapies [91,141]. These observations have highlighted adrenergic signaling as a promising therapeutic target and provided a rationale for combining β-blockers with established treatment modalities.

Although fluoropyrimidine-based chemotherapy remains a mCRC management, its long-term efficacy is frequently limited by the development of treatment resistance, which affects a substantial proportion of patients [91,142]. The TME, characterized by hypoxia, extracellular acidosis, fibrosis, and immune dysfunction, plays a central role in this process and has emerged as an attractive target for combination therapies involving β-blockers [143].

Among the key mediators of hypoxia-associated resistance is carbonic anhydrase IX (CAIX), a transmembrane enzyme induced by HIF-1α. CAIX promotes intracellular pH homeostasis by catalyzing the reversible hydration of carbon dioxide while simultaneously contributing to extracellular acidification. This adaptation enables cancer cells to survive under hypoxic and metabolically stressful conditions while facilitating ECM degradation, invasion, and metastatic spread [143,144]. Because CAIX expression is largely restricted to hypoxic tumor tissues and is strongly associated with aggressive disease and treatment resistance, it has emerged as both a valuable biomarker and a potential therapeutic target [143,144].

Mutations in the *TP53* gene, detected in approximately 40–50% of CRC cases, further contribute to therapeutic resistance. Under physiological conditions, p53 promotes mitochondrial apoptosis through induction of cytochrome c release and caspase activation. Loss of p53 function impairs these mechanisms, facilitating tumor-cell survival and reducing sensitivity to chemotherapy and radiotherapy [145].

Adrenergic signaling may additionally contribute to radioresistance through activation of the cAMP/PKA/CREB pathway. Increased CREB phosphorylation promotes survival signaling, enhances cellular adaptation to stress, and attenuates radiation-induced cell death [145]. Moreover, chronic β-adrenergic stimulation activates additional prosurvival pathways, including EGFR/AKT/ERK1/2 signaling, further contributing to treatment resistance. Consequently, pharmacological inhibition of β-ARs may sensitize tumor cells to radiation by suppressing these signaling networks, restoring mitochondrial apoptotic responses, and overcoming TME-driven radioresistance [145].

Accumulating experimental evidence suggests that β-blockers, particularly PrOH, may enhance the efficacy of conventional anticancer therapies through multiple complementary mechanisms. These include suppression of hypoxia-driven signaling, modulation of tumor-cell survival pathways, inhibition of angiogenesis, improvement of antitumor immune responses, and restoration of treatment sensitivity [145]. Consequently, β-blockers have emerged as promising adjunctive agents capable of potentiating the therapeutic effects of chemotherapy and radiotherapy in CRC. Nevertheless, prospective clinical studies are required to determine the optimal integration of β-blockers into multimodal treatment strategies and to establish their clinical benefit in CRC patients [91,141,142,143,144,145].

The therapeutic potential of β-blockers appears to be further enhanced when combined with anti-inflammatory agents, particularly cyclooxygenase-2 (COX-2) inhibitors. This dual-targeting strategy simultaneously suppresses adrenergic and inflammatory signaling, two major drivers of tumor progression and immunosuppression within the TME. Clinical studies have suggested that such an approach may reduce the risk of disease recurrence and improve treatment outcomes [146,147].

Synergistic interactions have also been proposed between β-blockers and systemic chemotherapy. Agents such as CAP induce DNA damage and activate apoptotic pathways in cancer cells, whereas β-adrenergic blockade attenuates survival signaling and facilitates mitochondrial apoptosis. Consequently, β-blockers may enhance the cytotoxic effects of chemotherapy and improve treatment sensitivity in advanced CRC [91].

Beyond their potential antitumor activity, β-blockers may provide additional clinical benefits through their well-established cardioprotective properties. This aspect is particularly relevant in oncology, where chemotherapy and radiotherapy may increase the risk of cardiovascular complications, especially in elderly patients and individuals with pre-existing cardiac disease [91,148,149].

Collectively, these findings suggest that pharmacological inhibition of adrenergic signaling represents a promising and readily translatable therapeutic strategy for CRC. By modulating the TME, enhancing sensitivity to conventional therapies, and attenuating resistance to chemotherapy and radiotherapy, β-blockers may improve treatment efficacy and contribute to reducing recurrence risk and improving long-term DFS in CRC patients. Owing to their well-established safety profile, broad clinical availability, and low cost, β-blockers represent attractive candidates for drug repurposing and multimodal treatment strategies in CRC [121]. The key molecular targets and biological pathways underlying the potential antitumor effects of β-blockers in CRC are summarized in Table 2.

## 4. β-Blockers’ Potential Applications in Colorectal Cancer Therapy

### 4.1. Preclinical Studies

Despite substantial advances in CRC treatment, including surgery, chemotherapy, radiotherapy, targeted therapies, and immunotherapy, treatment resistance and therapy-related toxicities remain major clinical challenges. These limitations have stimulated growing interest in drug repurposing strategies aimed at identifying novel applications for approved medications with well-established safety profiles [18,151]. Among the agents currently being investigated, β-blockers have emerged as promising candidates because of their ability to modulate adrenergic signaling pathways involved in tumor progression, angiogenesis, metastasis, and immune evasion. This section summarizes recent preclinical studies evaluating the potential therapeutic role of β-blockers in CRC.

Importantly, the available preclinical evidence should be interpreted in the context of the marked molecular heterogeneity of CRC and the diversity of experimental models used to investigate β-adrenergic signaling. The Consensus Molecular Subtype (CMS) classification distinguishes CMS1, characterized by MSI and immune activation; CMS2, associated with WNT/MYC signaling; CMS3, linked to metabolic dysregulation and frequent KRAS mutations; and CMS4, characterized by stromal activation, angiogenesis, EMT, and poor prognosis [1]. Clinically relevant biomarkers, including KRAS/NRAS mutations, BRAF^V600E^, MSI/dMMR status, and HER2 amplification, influence treatment selection and therapeutic response [1,27,36,37,38,39,41,42,109]. Although their predictive value for β-blocker therapy remains unknown, subtype-specific differences in signaling activity, immune composition, and TME characteristics may influence sensitivity to β-adrenergic blockade. Moreover, not all mechanisms discussed in this review have been directly demonstrated in CRC, and in several cases the proposed interactions are supported by evidence from other tumor models or experimental systems. Therefore, these findings should be interpreted with appropriate caution until validated in CRC. Nevertheless, the particularly strong response to PrOH observed in BRAF-mutated HT-29 cells suggests that tumor molecular characteristics may influence therapeutic responsiveness to β-blockade [121,124]. Future studies should therefore evaluate β-blockers in molecularly characterized CRC models to identify patient subgroups most likely to benefit from this therapeutic strategy.

A growing body of experimental evidence suggests that β-blockers may represent a safe, cost-effective, and readily available adjunctive strategy for CRC treatment. One of the most important clinical challenges in CRC management is resistance to fluoropyrimidine-based chemotherapy, particularly 5-FU, which remains a cornerstone of systemic treatment [91,152]. Because therapeutic options for patients with resistant disease remain limited, considerable attention has been directed toward identifying agents capable of restoring treatment sensitivity.

A notable example is the study by Puzderova et al. [91], which investigated the potential of PrOH to overcome 5-FU resistance through modulation of the TME. Using two-dimensional (2D) and three-dimensional (3D) cell culture models, as well as in vivo experimental systems, the authors demonstrated that PrOH effectively inhibits β-adrenergic signaling and counteracts adaptive responses to tumor hypoxia. In particular, treatment with PrOH significantly reduced the expression of HIF-1α and CAIX, two key mediators of cancer-cell survival under hypoxic conditions and important regulators of extracellular acidification within the TME. As a consequence, PrOH acted as a potent chemosensitizing agent, reducing tumor growth and migratory capacity while restoring sensitivity to 5-FU in resistant CRC models [91].

These findings provide strong preclinical evidence that β-blockers may enhance the efficacy of conventional chemotherapy by targeting hypoxia-driven resistance mechanisms and disrupting TME-mediated survival pathways.

Further evidence supporting the antitumor potential of PrOH was provided by Barathova et al. [62], who demonstrated that PrOH reduces the metastatic potential, viability, and proliferative capacity of CRC cells cultured as multicellular spheroids. β-AR blockade disrupted adaptive responses to hypoxia by reducing the expression of HIF-1α and CAIX, resulting in partial normalization of the TME and impairment of cellular homeostasis. Furthermore, PrOH inhibited mitochondrial metabolism, thereby limiting the energy supply required for tumor growth, invasion, and migration. Collectively, these findings indicate that PrOH may weaken tumor adaptive mechanisms and increase susceptibility to anticancer therapies [77].

Additional support for β-blocker-based therapeutic strategies was provided by Weng et al. [137], who developed copper–propranolol nanoparticles (Cu-PN) as a dual-function nanoplatform designed to enhance immunotherapy efficacy in CRC. This system enables the simultaneous delivery of copper ions and PrOH, thereby combining copper-dependent cell death induction with β-AR blockade. The resulting immunogenic cell death (ICD) was accompanied by reversal of T-cell exhaustion and restoration of effector immune functions. In vitro experiments performed using CT26 CRC cells and patient-derived organoids demonstrated enhanced cytotoxicity and increased ICD induction compared with monotherapy. In vivo, Cu-PN nanoparticles significantly inhibited tumor growth and remodeled the TME through enhanced dendritic-cell maturation, increased cytokine production, and activation of CD8^+^ T lymphocytes. Notably, combination treatment with anti-PD-1 therapies further improved antitumor efficacy, highlighting the potential of Cu-PN nanoparticles as a novel immuno-oncology strategy for CRC [137].

Alzahrani et al. [121,124] provided compelling evidence supporting the therapeutic potential of PrOH in CRC. Using human CRC cell lines (HCT-116, HT-29, SW-480, and SW-620), the authors demonstrated that PrOH significantly inhibited cell proliferation, migration, and colony formation, with the strongest effects observed in the BRAF-mutated HT-29 cell line. Treatment with PrOH induced apoptosis and cell-cycle arrest, thereby reducing tumor aggressiveness and metastatic potential. Furthermore, combination treatment with CAP enhanced antitumor activity in metastatic SW-620 cells, suggesting that PrOH may function not only as a single agent but also as a valuable component of combination treatment strategies aimed at overcoming therapeutic resistance in CRC [121].

In a subsequent study, the same research group further investigated the effects of PrOH, both as monotherapy and in combination with CAP, in HCT-116 and HT-29 CRC cells [124]. The authors demonstrated that combined treatment significantly reduced cell viability, particularly in *BRAF*-mutated HT-29 cells, through the induction of ferroptosis, an iron-dependent form of regulated cell death. Importantly, activation of ferroptotic pathways may overcome the apoptosis resistance frequently observed in aggressive CRC phenotypes. Moreover, ferroptosis induction was accompanied by activation of necroptotic signaling pathways, indicating the simultaneous engagement of multiple regulated cell-death mechanisms. Combination treatment also inhibited cell migration and enhanced antitumor immune responses, further supporting the role of PrOH as a synergistic adjuvant to CAP capable of overcoming chemoresistance in CRC [124].

Chronic psychological stress has been proposed as a factor that may promote CRC progression through sustained activation of the sympathetic nervous system and increased release of catecholamines, particularly Epi and NE. Experimental studies, including CRC models, indicate that these mediators activate β-ARs expressed by tumor, stromal, endothelial, and immune cells, thereby promoting prosurvival signaling, angiogenesis, EMT, and suppression of antitumor immunity [17,57,138]. However, some of the molecular mechanisms linking β-adrenergic signaling to these processes have been characterized primarily in other tumor types and therefore require further validation in CRC. Thus, chronic stress is currently regarded as a potential modifier of tumor progression rather than a direct cause of CRC, providing a biological rationale for investigating β-adrenergic blockade as a potential therapeutic strategy.

Preclinical studies conducted in animal models have further demonstrated a strong association between chronic adrenergic signaling and accelerated tumor progression. Qiao et al. [138] showed that suppression of chronic adrenergic signaling, achieved either through PrOH-mediated β-AR blockade or genetic deletion of β-ARs, significantly inhibited tumor growth in mouse models. Mechanistically, chronic stress impaired TCR activation and promoted the accumulation of terminally exhausted T cells within the TME. In contrast, β-adrenergic blockade restored immune homeostasis by reducing the proportion of terminally exhausted T cells while promoting the expansion of progenitor exhausted T-cell populations associated with durable antitumor immunity [138].

Similarly, Fjæstad et al. [93] demonstrated that oral administration of PrOH significantly delayed tumor progression in the MC38 murine CRC model and prolonged OS. The antitumor activity of PrOH was attributed to its multidirectional effects on the TME, including suppression of angiogenesis and enhancement of antitumor immune responses. In particular, PrOH promoted the recruitment of T lymphocytes into tumor tissue while simultaneously reducing the abundance of MDSCs, thereby weakening the immunosuppressive barrier and facilitating more effective immune-mediated tumor control [93].

Complementary evidence highlights the impact of PrOH on intracellular signaling pathways and its potential translational relevance. Liao et al. [61] demonstrated in the CT26WT murine CRC model that PrOH significantly reduced tumor growth through inhibition of the AKT/MAPK signaling cascade, as evidenced by decreased expression of phosphorylated AKT (p-AKT), ERK (p-ERK), and MEK (p-MEK). Importantly, these preclinical findings were supported by clinical observations showing that short-term preoperative administration of PrOH to CRC patients was associated with reduced p-ERK levels and enhanced CD8^+^ T-cell-mediated antitumor immune responses [61].

The synergistic potential of PrOH within drug-repositioning strategies was further explored by Anselmino et al. [153], who investigated its interaction with metformin. The authors demonstrated that combined treatment significantly enhanced antitumor activity in CRC models. In vitro studies revealed reduced cell viability and clonogenic capacity, increased apoptosis, and inhibition of both migratory potential and EMT. These effects were recapitulated in vivo, where combined treatment significantly reduced tumor burden while maintaining a favorable safety profile. Notably, the combination retained efficacy in 5-FU-resistant models, suggesting that PrOH may serve as a valuable component of therapeutic strategies targeting chemoresistant CRC [153,154].

More recently, Anselmino et al. [154] evaluated the combination of PrOH with chloroquine (CQ), another repurposed drug with recognized anticancer properties. In vitro analyses performed in CT26, HCT116, and HT29 cell lines demonstrated that both agents reduced cancer-cell viability in a dose-dependent manner while exerting minimal effects on non-malignant cells. Importantly, combined treatment produced significantly greater antitumor activity than either agent alone, resulting in enhanced inhibition of proliferation, increased apoptosis, and reduced migratory and clonogenic potential. These effects became more pronounced with prolonged drug exposure. Consistent with the in vitro findings, in vivo studies demonstrated that the PrOH–CQ combination significantly inhibited primary tumor growth and metastatic dissemination. Collectively, these findings indicate that PrOH-based combination therapies may represent a promising therapeutic approach for CRC and further support the rationale for integrating β-blockers into drug-repositioning strategies [153].

Additional evidence supporting the therapeutic versatility of PrOH was provided by Hu et al. [155], who investigated its combination with the oncolytic virus T1012G in CRC models. The combined treatment produced a synergistic cytotoxic effect against CRC cells and induced sustained tumor regression in an HCT116 xenograft model. Mechanistically, the enhanced antitumor activity was associated with increased apoptosis, as demonstrated by elevated levels of cleaved caspase 3. Furthermore, combination therapy exerted a pronounced anti-angiogenic effect through suppression of VEGF secretion, with the greatest inhibition observed in the dual-treatment group. Importantly, PrOH did not interfere with viral replication, indicating that its therapeutic contribution was primarily related to modulation of the TME and sensitization of tumor cells to virus-induced cell death. These findings suggest that PrOH may represent a valuable adjunct to oncolytic virotherapy and support further investigation of this strategy in clinical settings [155].

Despite the substantial body of evidence supporting the antitumor activity of PrOH, recent studies have also explored the therapeutic potential of its metabolites and stereoisomers. Yi et al. [74] compared the effects of PrOH, 4-hydroxypropranolol, and PrOH enantiomers on CRC progression. All tested compounds inhibited CRC-cell viability in a time- and concentration-dependent manner and significantly suppressed tumor growth in vivo. Mechanistic analyses revealed inhibition of the Akt/MAPK signaling pathways together with modulation of the TME through effects on immune checkpoint molecules, including PD-1. Notably, 4-hydroxypropranolol demonstrated the strongest antitumor activity among all tested compounds, suggesting superior tumor-suppressive properties compared with PrOH and its enantiomers. The authors proposed that this enhanced efficacy may be related to a higher affinity of 4-hydroxypropranolol for β_2_-ARs. These findings identify 4-hydroxypropranolol as a promising candidate for further investigation as a potential therapeutic agent in CRC [74].

Collectively, preclinical studies consistently demonstrate that PrOH exerts antitumor effects through multiple complementary mechanisms, including inhibition of β-adrenergic signaling, suppression of angiogenesis, modulation of antitumor immunity, induction of apoptosis and ferroptosis, attenuation of hypoxia-associated adaptation, and enhancement of sensitivity to conventional anticancer therapies. Although these findings provide strong biological support for the repositioning of PrOH in CRC, further translational and clinical studies are required to determine the extent to which these preclinical benefits can be translated into meaningful improvements in patient outcomes.

### 4.2. Clinical Trials

Although numerous novel therapeutic strategies are currently being investigated for CRC, only a limited number have been successfully translated into routine clinical practice [156]. Similarly, despite encouraging preclinical evidence supporting the antitumor activity of β-blockers, clinical data remain limited and are derived predominantly from retrospective and observational studies. Although several retrospective analyses have reported an association between β-blocker use and improved survival outcomes in patients with CRC, the available evidence is insufficient to establish a causal relationship or support their routine clinical use [102]. Nevertheless, these promising preclinical findings have stimulated growing interest in evaluating β-blockers as potential adjunctive agents in clinical oncology. The clinical studies discussed below should therefore be interpreted in the context of their methodological limitations and the current lack of prospective randomized evidence. This section summarizes the most relevant clinical studies investigating the potential role of β-blockers in CRC management.

One of the earliest clinical observations suggesting a potential benefit of β-blockers in CRC was reported by Fiala et al. [157]. In this retrospective single-center study, incidental β-blocker use was associated with improved progression-free survival (PFS) and OS in patients with mCRC receiving bevacizumab-based therapy. The authors proposed that β-AR blockade may enhance the efficacy of anti-VEGF treatment by attenuating stress-induced tumor-cell proliferation, angiogenesis, and metastatic dissemination. Based on these findings, β-blockers were suggested as potentially preferable antihypertensive agents in this patient population. However, the retrospective design and the potential influence of residual confounding preclude definitive conclusions, and prospective studies are required to validate these observations [157].

More direct clinical evidence was provided by the randomized clinical trial NCT00888797 conducted by Haldar [53]. In this study, patients with CRC received PrOH in combination with the COX-2 inhibitor etodolac for 20 days during the perioperative period. This intervention was designed to counteract the pro-tumorigenic effects of surgical stress, which are known to promote inflammation, immunosuppression, and metastatic dissemination. Molecular analyses of resected tumor specimens demonstrated significant modulation of the TME, including suppression of EMT, increased infiltration of NK cells, and reduced infiltration of monocytes and B lymphocytes [53].

These biological changes were accompanied by encouraging clinical observations. Analysis of the 3-year follow-up data demonstrated that no disease recurrence was observed among patients who fully adhered to the treatment protocol, whereas recurrence approached 30% in the placebo group [146]. Long-term follow-up reported by Ricon-Becker et al. [146] further supported the persistence of these findings. After five years of observation, no recurrence was reported in the treatment group, whereas the recurrence rate in the placebo group increased to approximately 50% [146].

Although these findings were obtained in a relatively small patient cohort, they provide preliminary clinical support for the hypothesis that perioperative modulation of adrenergic and inflammatory signaling may influence disease progression. Collectively, these studies suggest that targeted β-AR blockade during the perioperative “window of opportunity” may represent a promising strategy for reducing metastatic dissemination and improving long-term oncological outcomes in patients with CRC. However, these findings should be interpreted with caution because the available evidence is based on a limited number of patients, and confirmation in larger, adequately powered multicenter randomized clinical trials is required before this approach can be considered for routine clinical implementation [53,146].

Building on these preliminary findings, a larger multicenter validation study is currently underway within the randomized phase II clinical trial NCT03919461 [147,158]. This study includes a substantially larger patient cohort than the original trial and employs the same pharmacological protocol, consisting of a 20-day perioperative combination of PrOH and etodolac. Its primary objective is to determine whether the favorable alterations in TME and immune biomarkers observed previously translate into clinically meaningful improvements in 3-year DFS and 5-year OS. The results of this ongoing trial are expected to provide more robust evidence regarding the clinical utility of perioperative β-adrenergic blockade in CRC and will help clarify whether the encouraging biological effects observed to date translate into clinically meaningful patient benefit [147,158].

Additional evidence is provided by large population-based observational studies. Two complementary nationwide cohort studies conducted in Sweden suggested a potential protective association between perioperative β-blocker use and clinical outcomes in patients undergoing surgery for CRC. In patients undergoing emergency surgery, preoperative β-blocker therapy was associated with a 60% reduction in 1-year mortality (HR = 0.40), and this association persisted even among individuals who developed severe postoperative complications [159]. Similarly, in a multicenter cohort study of patients undergoing elective CRC surgery, Ahl et al. [160] reported improved survival outcomes despite the older age and greater comorbidity burden of β-blocker users. Specifically, β-blocker therapy was associated with a 43% reduction in all-cause mortality within one year (HR = 0.57) and a significant reduction in CRC-specific mortality during five years of follow-up (HR = 0.80) [160]. Although the consistency of these findings across different surgical settings supports the hypothesis that attenuation of perioperative adrenergic hyperactivation may improve postoperative and long-term oncological outcomes, both studies were observational in nature and therefore remain susceptible to residual confounding despite statistical adjustment. Consequently, these findings should be interpreted cautiously and warrant confirmation in prospective randomized clinical trials [159,160].

Because most CRC cases arise from premalignant lesions, particularly adenomatous and serrated polyps, the potential role of β-blockers in chemoprevention has also attracted increasing attention [161]. In a large population-based Swedish cohort study, Emilsson et al. [162] reported that initiation of β-blocker therapy within two years after the diagnosis of colorectal polyps was associated with a reduced risk of subsequent CRC development. This association appeared to be more pronounced among women, in whom β-blocker use was also associated with lower CRC-specific mortality [162]. However, these findings should be interpreted cautiously because of the observational nature of the study and the potential influence of residual confounding. Consequently, although the results suggest that β-blockers may influence the early stages of colorectal carcinogenesis, they do not establish a causal relationship and require confirmation in prospective studies specifically designed to evaluate chemopreventive efficacy. Nevertheless, these observations support further investigation of β-blockers as potential chemopreventive agents in high-risk populations [162]. A comparative summary of representative clinical studies evaluating β-blocker therapy in CRC is presented in Table 3.

Because the included studies evaluated different β-blockers with distinct receptor selectivity, doses, treatment durations, and concomitant anticancer therapies, direct comparison of their clinical outcomes should be interpreted with caution. Overall, the available clinical evidence suggests that perioperative PrOH-based strategies and incidental β-blocker use may be associated with improved oncological outcomes in selected patient populations. However, these findings are derived predominantly from retrospective observational studies and relatively small clinical trials involving heterogeneous patient populations. Moreover, differences in β-blocker exposure, receptor selectivity, treatment duration, and concomitant therapies, together with the potential for residual confounding resulting from the cardiovascular indications for β-blocker therapy, limit the interpretation of the available evidence. Consequently, although several studies have reported associations between β-blocker use and improved survival or reduced recurrence, a causal relationship has not yet been established. Furthermore, the biological and potentially oncological effects of cardioselective and non-selective β-blockers may differ because of differences in β_2_-AR blockade, further complicating comparisons across studies. Therefore, large, adequately powered prospective randomized clinical trials are required to determine whether β-blockers provide clinically meaningful benefit and can be recommended as adjunctive therapy for patients with CRC.

### 4.3. Limitations and Barriers to Clinical Implementation of β-Blockers in Colorectal Cancer

Given their low cost, well-established cardiovascular safety profile, and widespread clinical availability, β-blockers represent attractive candidates for drug repurposing in CRC therapy [102]. Nevertheless, despite encouraging preclinical and emerging clinical evidence, their integration into routine oncological practice remains limited by several important challenges, including the restricted translational value of preclinical models, the predominance of observational clinical studies, substantial methodological heterogeneity, uncertainties regarding clinically relevant dosing, and the lack of standardized molecular monitoring strategies.

While β-blockers can counteract the proliferation-promoting effects of catecholamines, their therapeutic efficacy is likely to depend on the expression profile and density of β_1_-AR and β_2_-AR in tumor cells and within the TME. Second-generation β-blockers, which exhibit selective affinity for β_1_-AR, minimize adverse effects associated with β_2_-AR blockade and are therefore preferred for the treatment of cardiovascular diseases. However, this receptor selectivity may also limit their antitumor efficacy if β_2_-adrenergic signaling is the predominant driver of angiogenesis, invasion, and metastasis in CRC. Conversely, third-generation β-blockers possess additional vasodilatory properties that may increase the risk of hemodynamic complications, including hypotension, particularly in patients receiving intensive anticancer therapy. Furthermore, β-blocker therapy is unlikely to be universally effective, as its therapeutic benefit may depend on tumor molecular characteristics, β-AR expression, and patient-specific biological factors [15].

Although numerous in vitro and in vivo studies have demonstrated promising antitumor effects of β-blockers, these experimental models do not fully recapitulate the complexity of the human TME. The TME comprises not only malignant cells but also stromal components, diverse immune-cell populations, ECM, and dynamic biochemical and biomechanical interactions. Consequently, the simplified nature of experimental systems may partially explain the discrepancy between encouraging preclinical findings and the more heterogeneous outcomes observed in clinical investigations. These limitations underscore the need for more sophisticated translational models and well-designed prospective clinical trials that more accurately reflect patient-specific tumor biology and facilitate the translation of preclinical findings into clinical practice [147].

An additional translational challenge concerns the considerable discrepancy between β-blocker concentrations used in experimental models and clinically achievable drug exposure in humans. Many in vitro studies employ concentrations that exceed therapeutic plasma levels by one to two orders of magnitude, whereas some animal studies use doses substantially higher than those routinely administered for cardiovascular indications. Consequently, caution is warranted when extrapolating these findings to clinical practice. To illustrate this issue, Table 4 summarizes representative β-blocker doses reported in preclinical and clinical studies and compares them with clinically approved human dosing regimens.

Another major limitation of the current evidence is that most available clinical data are derived from observational studies. These studies are inherently susceptible to indication bias, residual confounding, and selection bias. Moreover, clinical conditions commonly requiring β-blocker therapy, including hypertension, coronary artery disease, and heart failure, may independently influence cancer outcomes and survival, thereby complicating the causal interpretation of the observed associations [102]. Consequently, establishing a causal relationship between β-blocker use and improved oncological outcomes remains challenging, highlighting the need for adequately powered randomized controlled trials.

Interpretation of the available clinical evidence is further complicated by considerable methodological heterogeneity among published studies. Differences in patient populations, tumor stage, treatment regimens, surgical approaches, use of targeted therapies (e.g., bevacizumab), β-blocker class, dosage, treatment duration, endpoint definitions, and follow-up periods may all substantially influence clinical outcomes [102,147,157]. In addition, several studies have been limited by relatively small sample sizes and insufficient statistical power for subgroup analyses, necessitating caution when extrapolating their findings to broader CRC populations.

Even the most promising perioperative studies investigating PrOH in combination with etodolac have important limitations, including moderate sample sizes, limited assessment of long-term safety, and the exclusion of patients with significant cardiovascular contraindications [147]. Consequently, the generalizability of these findings remains uncertain and requires confirmation in larger multicenter randomized clinical trials.

Future clinical studies should incorporate comprehensive molecular monitoring strategies to better characterize treatment responses and identify patients most likely to benefit from β-blocker therapy. Integration of preoperative tumor biopsies with serial liquid biopsy analyses, including circulating tumor DNA (ctDNA), extracellular vesicles, and exosomal biomarkers, may provide valuable insights into the biological effects of β-blocker therapy, facilitate biomarker-guided patient selection, and improve the design of future precision oncology trials [53].

Another important unresolved issue concerns patient selection. Although increasing evidence suggests that β-adrenergic signaling contributes to CRC progression, the predictive value of β_1_-AR and β_2_-AR expression, molecular subtype, immune status, and stress-related biomarkers remains poorly defined. Consequently, it is currently unclear which patients are most likely to benefit from β-blocker therapy. Identification and prospective validation of predictive biomarkers should therefore represent a major priority for future translational and clinical research.

Ultimately, the successful implementation of β-blockers in CRC management will depend on the results of adequately powered prospective randomized clinical trials capable of confirming both efficacy and safety. Validation of perioperative and long-term treatment protocols, together with the identification of predictive biomarkers, will be essential for defining the role of β-blockers within personalized CRC therapy. Until such evidence becomes available, β-blockers should be regarded as promising investigational agents rather than components of routine clinical management. Their future incorporation into multimodal treatment strategies will depend on robust clinical evidence demonstrating meaningful therapeutic benefit in carefully selected patient populations.

### 4.4. Adverse Effects and Contraindications to β-Blocker Therapy

Due to the widespread distribution of β-ARs throughout the body and their involvement in numerous physiological processes, β-blocker therapy is associated with a broad spectrum of potential adverse effects. The most common include bradycardia and hypotension, which result from reduced heart rate and myocardial contractility. Patients may also experience fatigue, dizziness, exercise intolerance, and gastrointestinal symptoms such as nausea or constipation. In addition, β-blockers have been associated with sexual dysfunction, including erectile dysfunction, although the reported incidence varies among different agents [51,164].

The safety profile of β-blockers depends largely on their pharmacodynamic and pharmacokinetic properties, particularly receptor selectivity, intrinsic sympathomimetic activity, lipophilicity, and metabolic characteristics. Cardioselective β_1_-blockers generally exhibit a lower incidence of bronchospasm and peripheral vasoconstriction than non-selective agents, making them preferable for many patients with cardiovascular disease [51,164].

Bisoprolol, for example, has been shown to cause fatigue more frequently than placebo, primarily because of its physiological effects on heart rate and cardiac output. Importantly, no significant increase in bronchospasm or worsening of respiratory symptoms was observed in the analyzed studies, reflecting its high selectivity for β_1_-AR. Other adverse effects, including dizziness, headache, and occasional sexual dysfunction, were generally mild and transient, allowing effective blood pressure control in most patients [164].

Lipophilic β-blockers may also induce central nervous system adverse effects because of their ability to cross the blood–brain barrier. Patients receiving these agents may experience insomnia, sleep disturbances, vivid dreams, nightmares, depression, or fatigue. Evidence suggests that PrOH, one of the most lipophilic β-blockers, is associated with a higher risk of neuropsychiatric adverse effects than many other antihypertensive agents [165]. Consequently, the pharmacokinetic properties of individual β-blockers should be considered when selecting the most appropriate agent for long-term therapy [51,165].

β-Blockers may also influence glucose metabolism by impairing glycogenolysis and masking the adrenergic manifestations of hypoglycemia, particularly tachycardia and tremor. Therefore, careful monitoring is recommended in patients with diabetes mellitus receiving insulin or insulin secretagogues. Furthermore, some β-blockers have been associated with modest weight gain and reduced exercise tolerance, particularly during the initial phase of treatment [51].

Although asthma was historically considered an absolute contraindication to β-blocker therapy, current clinical guidelines permit the cautious use of cardioselective β_1_-blockers in selected patients with mild or moderate obstructive airway disease when clinically indicated, whereas non-selective β-blockers should generally be avoided because of the increased risk of bronchospasm [51,166]. Acute decompensated heart failure, severe bradycardia, advanced atrioventricular block in the absence of a pacemaker, symptomatic hypotension, and cardiogenic shock remain important contraindications or situations requiring particular caution. Drug selection should also take patient-specific comorbidities into account. For example, sotalol is contraindicated in patients with congenital long QT syndrome because of its potassium channel-blocking properties and the associated risk of ventricular arrhythmias, whereas β-blockers should generally be avoided or used with caution in patients with severe Raynaud’s phenomenon because they may aggravate peripheral vasoconstriction [51,166].

Overall, β-blockers are generally well tolerated and possess a well-established safety profile when prescribed according to current clinical guidelines. Nevertheless, careful patient selection, appropriate drug choice, dose titration, and close monitoring remain essential, particularly when β-blockers are considered as adjunctive agents in patients undergoing multimodal anticancer therapy, who frequently present with multiple comorbidities and increased susceptibility to treatment-related adverse events.

## 5. Future Perspectives

The growing body of evidence supporting the antitumor activity of β-blockers has generated considerable interest in their potential application as adjunctive agents in CRC therapy. Nevertheless, several important challenges remain to be addressed before β-blockers can be successfully integrated into routine clinical practice. Future research should focus on identifying predictive biomarkers, optimizing treatment strategies, and generating robust clinical evidence to support their implementation in precision oncology.

One of the most promising directions involves the identification of predictive biomarkers that could facilitate patient stratification and improve treatment selection. Since β-adrenergic signaling is mediated primarily through β_1_- and β_2_-ARs, characterization of receptor expression patterns in tumor cells and components of the TME may help identify patients most likely to benefit from β-blocker therapy. In addition, molecular biomarkers associated with angiogenesis, inflammation, stress response, and immune regulation may provide valuable information regarding treatment responsiveness and support biomarker-guided therapeutic decision-making.

Another important area of investigation is the integration of β-blockers with existing anticancer treatment modalities. Accumulating evidence suggests that β-blockers may enhance the efficacy of chemotherapy, targeted therapies, and immunotherapy by modulating tumor-cell signaling pathways and remodeling the TME. In particular, the combination of β-blockers with immune checkpoint inhibitors represents a promising therapeutic strategy, as inhibition of β-adrenergic signaling may alleviate immunosuppression, increase cytotoxic T-cell infiltration, and enhance antitumor immune responses. Future studies should determine the optimal treatment combinations, sequencing strategies, and treatment duration required to maximize clinical benefit while minimizing toxicity.

The perioperative period has also emerged as a promising therapeutic window for β-blocker administration. Recent perioperative studies suggest that short-term treatment with PrOH, particularly in combination with anti-inflammatory agents such as etodolac, may attenuate surgery-induced stress responses, reduce prometastatic signaling, and favorably modulate the TME. However, adequately powered prospective randomized clinical trials are required to determine whether perioperative β-blockade can improve long-term oncological outcomes in patients with CRC.

The development of advanced molecular monitoring approaches is expected to further accelerate the clinical translation of β-blockers. Liquid biopsy technologies, including ctDNA, extracellular vesicles, exosomal biomarkers, and circulating immune-cell profiling, may enable real-time assessment of treatment response, facilitate early identification of responders and non-responders, and support personalized therapeutic decision-making.

Recent advances in multi-omics technologies and patient-derived organoid models are expected to further accelerate the clinical translation of β-blockers in CRC. Integration of genomic, transcriptomic, proteomic, metabolomic, and spatial transcriptomic data may improve biomarker discovery, facilitate patient stratification, and identify molecular determinants of treatment response. Furthermore, patient-derived organoids, organ-on-chip technologies, and advanced three-dimensional tumor models may provide clinically relevant platforms for evaluating β-blocker-based combination therapies, improving prediction of therapeutic responses, and accelerating their translation into clinical practice.

In addition to PrOH, growing attention has been directed toward the development of novel β-blocker derivatives and metabolites with enhanced anticancer activity. Recent studies have demonstrated that 4-hydroxypropranolol may exhibit stronger antitumor activity than PrOH itself, suggesting that further investigation of β-blocker stereoisomers and metabolites may identify more effective compounds for oncological applications. Future studies should clarify their molecular mechanisms of action, pharmacological properties, and therapeutic potential in CRC.

Collectively, these future directions highlight the transition of β-blockers from conventional cardiovascular drugs toward promising components of precision oncology. Their successful clinical implementation will depend on biomarker-guided patient selection, advanced molecular profiling, innovative preclinical models, rational combination therapies, and well-designed prospective randomized clinical trials. If these challenges can be successfully addressed, β-blockers may become valuable adjunctive agents in personalized CRC therapy, providing a cost-effective, biologically rational, and clinically feasible strategy for improving patient outcomes.

## 6. Conclusions

CRC remains a major therapeutic challenge despite substantial advances in surgery, chemotherapy, targeted therapy, and immunotherapy. Growing evidence indicates that stress-mediated β-adrenergic signaling plays an important role in CRC progression by promoting tumor cell survival, angiogenesis, metastatic dissemination, immune evasion, and remodeling of the TME. Consequently, this pathway has emerged as a promising target for therapeutic intervention. The available preclinical and clinical evidence reviewed in this article suggests that β-blockers, particularly PrOH, may exert multifaceted antitumor effects through modulation of tumor cell survival pathways, inhibition of angiogenesis and metastatic dissemination, remodeling of the TME, and enhancement of antitumor immune responses.

In addition to their direct anticancer effects, β-blockers may enhance the efficacy of conventional anticancer therapies, including chemotherapy, targeted therapies, and immunotherapy. Their well-established clinical safety profile, widespread availability, and relatively low cost make them attractive candidates for drug repurposing. Nevertheless, the current body of evidence remains largely derived from preclinical studies, retrospective analyses, and a limited number of clinical investigations.

At present, the available clinical evidence is insufficient to support the routine implementation of β-blockers in the management of CRC. Further prospective, well-designed randomized clinical trials are required to establish their clinical efficacy, determine optimal treatment regimens, identify predictive biomarkers for patient selection, and evaluate their long-term safety when used in combination with standard anticancer therapies.

Overall, β-blockers represent a biologically plausible therapeutic strategy that merits further investigation rather than routine clinical application at the present time. Continued translational research together with rigorously designed prospective clinical trials will be essential to determine whether β-blockers can ultimately be incorporated into multimodal treatment strategies for carefully selected patients with CRC.

## Figures and Tables

**Table 1:** Current treatment modalities in colorectal cancer: principal therapeutic approaches, clinical applications, advantages, and limitations.

Treatment Modality	Main Approaches	Clinical Use in CRC	Advantages	Limitations	References
Surgical treatment	Open, laparoscopic, robotic, navigation-guided and endoscopic surgery; ablative techniques	Standard treatment for localized and resectable CRC; selected metastatic lesions	Potentially curative; improved precision and faster recovery with minimally invasive approaches	Postoperative complications, risk of recurrence, and surgery-related morbidity	[19,20,23,24,25,26]
Chemotherapy	Fluoropyrimidine-based monotherapy and combination regimens (FOLFOX, FOLFIRI, CAPOX, XELIRI, FOLFOXIRI)	Perioperative, locally advanced, and metastatic CRC treatment	Improved survival, disease control, and reduced recurrence risk	Systemic toxicity, adverse effects, treatment resistance, and impaired quality of life	[20,21,28,29,30,31]
Radiotherapy	External-beam radiotherapy, stereotactic radiotherapy, IORT, and brachytherapy	Local control and neoadjuvant treatment, mainly in patients at high risk of local recurrence	Tumor downstaging, improved resectability, and targeted local tumor control	Risk of damage to healthy tissues, toxicity, chronic radiation enteritis, fertility impairment	[12,19,20,32,34]
Targeted therapy	Anti-EGFR, anti-VEGF, and anti-HER2 therapies	Personalized treatment of metastatic CRC with specific molecular profiles	Selective antitumor activity, improved survival, and better disease control	Treatment-related toxicities and resistance	[5,16,19,20]
Immunotherapy	Immune checkpoint inhibitors, (ACT, TILs, CAR-T, CAR-NK, TCR-T), and therapeutic cancer vaccines	Primarily metastatic MSI-H/dMMR CRC; emerging strategies for MSS/pMMR CRC	Durable antitumor responses and personalized immunotherapy	Limited efficacy in MSS/pMMR CRC and immune-related toxicities	[21,22,23,24,25]

**Abbreviations:** ACT—adoptive cell therapy; CAR-NK—chimeric antigen receptor natural killer cell therapy; CAR-T—chimeric antigen receptor T-cell therapy; CRC—colorectal cancer; dMMR—deficient mismatch repair; EGFR—epidermal growth factor receptor; HER2—human epidermal growth factor receptor 2; IORT—intraoperative radiotherapy; MSI-H—microsatellite instability-high; MSS—microsatellite stable; pMMR—proficient mismatch repair; TCR-T—T-cell receptor-engineered T-cell therapy; TILs—tumor-infiltrating lymphocytes; VEGF—vascular endothelial growth factor.

**Table 2:** Molecular mechanisms underlying the potential antitumor effects of β-blockers in colorectal cancer.

Mechanism	Key Molecular Targets	Biological Effects	References
β2-AR/cAMP/PKA/CREB signaling	β2-AR, cAMP, PKA, CREB, ATF	↓ proliferation, ↓ survival, ↓ angiogenesis, ↑ apoptosis	[47,54,55,56]
PI3K/Akt/mTOR signaling	PI3K, Akt, mTOR, cyclin D1	↓ tumor growth, ↓ cell survival, ↓ treatment resistance	[56,57,67]
RAS/RAF/MEK/ERK signaling	RAS, RAF, MEK, ERK	↓ proliferation, ↓ cell-cycle progression, ↓ metastatic potential	[64,65,70,74]
Angiogenesis and hypoxia-associated signaling	VEGF, VEGFA, HIF-1α	↓ angiogenesis, ↓ hypoxia adaptation, ↑ vessel normalization	[15,89,90,91,92,93,95]
EMT and metastasis	TGF-β, MMPs, CEBPB, TRIM2, p53	↓ invasion, ↓ migration, ↓ metastatic dissemination	[57,108,109,110,111]
Apoptosis	BCL-2, BAX, caspase-3	↑ apoptosis, ↑ treatment sensitivity	[115,116,118]
Immune modulation and immunotherapy	Arginase 1, IDO, PD-1, PD-L1, CTLA-4,	↓ MDSCs/TAMs, ↑ CD8^+^ T cells activity, ↑ immunotherapy response	[93,127,128,129,130,131,150]
Synergy with conventional therapies	CAIX, EGFR, AKT, ERK	↑ chemotherapy response, ↑ radiosensitivity	[143,144,145]

**Symbols:** ↑ and ↓ denote increased and decreased expression or biological activity, respectively. **Abbreviations:** AKT—protein kinase B; ATF—activating transcription factor; β_2_-AR—beta-2 adrenergic receptor; BAX—BCL2-associated X protein; BCL-2—B-cell lymphoma 2 protein; CAIX—carbonic anhydrase IX; cAMP—cyclic adenosine monophosphate; CEBPB—CCAAT/enhancer-binding protein beta; CREB—cAMP response element-binding protein; CTLA-4—cytotoxic T-lymphocyte-associated protein 4; EGFR—epidermal growth factor receptor; EMT—epithelial–mesenchymal transition; ERK—extracellular signal-regulated kinase; HIF-1α—hypoxia-inducible factor 1-alpha; IDO—indoleamine 2,3-dioxygenase; MDSCs—myeloid-derived suppressor cells; MEK—mitogen-activated protein kinase kinase; MMPs—matrix metalloproteinases; mTOR—mechanistic target of rapamycin; p53—tumor protein p53; PD-1—programmed cell death protein 1; PD-L1—programmed death-ligand 1; PI3K—phosphoinositide 3-kinase; PKA—protein kinase A; RAF—rapidly accelerated fibrosarcoma kinase; RAS—rat sarcoma GTPase; TAMs—tumor-associated macrophages; TGF-β—transforming growth factor beta; TRIM2—tripartite motif-containing 2; VEGF—vascular endothelial growth factor; VEGFA—vascular endothelial growth factor A.

**Table 3:** Summary of representative clinical studies evaluating β-blocker therapy in colorectal cancer, including study design, treatment characteristics, clinical outcomes, and major methodological limitations.

Study	Study Design	Disease Stage	Total N (β-Blocker Users)	β-Blocker (Type, Selectivity, Dose)	Treatment Duration	Concomitant Therapy	Adjusted Effect Estimates (95% CI)/Main Findings	Major Limitations	References
Fiala et al. (2019)	Retrospective single-center cohort	mCRC (Stage IV)	514 (126)	Cardioselective (n = 61) and non-selective (n = 65); standard cardiovascular doses (dose not stratified)	Chronic therapy before and during chemotherapy	Bevacizumab-based chemotherapy	PFS: HR = 0.76 (0.61–0.96); OS: HR 0.73 (0.56–0.95). β-blocker use was independently associated with longer PFS and OS in multivariable analysis.	Retrospective design, single-center, no stratification according to β-blocker type, dose, or receptor selectivity	[157]
Ahl et al. (2019)	Nationwide observational cohort study	CRC I-IV stage (41.9% Stage III)	3139 (671)	Unspecified β-blockers (predominantly β_1_-selective); standard cardiovascular doses	Preoperative chronic therapy	Standard emergency surgical care	1-year all-cause mortality: HR = 0.40 (0.21–0.78)	Observational design, potential unmeasured confounders, no information on treatment adherence, reliance on prescription registry data	[159]
Haldar et al. (2020)	Phase II double-blind placebo-controlled biomarker RCT (NCT00888797)	Resectable CRC (Stage I-III)	34 (16)	PrOH (non-selective β_1_/β_2_); 20–80 mg twice daily	20 days perioperative treatment	Etodolac, 400 mg twice daily	Reduced EMT; decreased tumor-infiltrating CD14^+^ monocytes and CD19^+^ B cells; increased CD56^+^ NK-cell infiltration; favorable modulation of GATA, STAT, EGR and CREB transcriptional activity. Three-year recurrence (exploratory): ITT 12.5% vs. 33.3% (*p* = 0.239); per-protocol 0% vs. 29.4% (*p* = 0.054).	outcome Small single-center biomarker RCT; exploratory clinical endpoints; limited statistical power; combined propranolol–etodolac intervention precludes attribution of effects to propranolol alone; not powered for survival outcomes.	[53]
Ricon-Becker et al. (2023)	Long-term follow-up of a Phase II double-blind placebo-controlled RCT (NCT00888797)	Resectable CRC (Stage I-III)	34 (16)	PrOH (non-selective β_1_/β_2_); 20–80 mg twice daily	20 days perioperative	Etodolac, 400 mg twice daily	5-year follow-up (per-protocol): recurrence 0/11 (0%) vs. 8/17 (47%) (*p* = 0.007); mortality 0/11 (0%) vs. 3/17 (17.6%) (*p* = 0.151). Eight-year follow-up: recurrence-free survival HR = 0.15 (95% CI 0.03–0.81).	Small single-center pilot RCT; limited statistical power and generalizability; combined PrOH-etodolac intervention limits attribution of effects	[146]
Emilsson et al. (2025)	Emulated target trial (nationwide retrospective cohort study)	Premalignant (colorectal polyps)	30,399 (2083)	Unspecified β-blockers; standard cardiovascular doses	Initiated within 2 yrs after polyp diagnosis	Standard care	Incident CRC HR = 0.87 (0.85–0.89) CRC mortality: HR = 0.96 (0.83–1.09)	Observational nature; residual unmeasured confounding; lack of detailed endoscopic and pathological data	[162]
Sakis et al. (2026) (COMPIT2 trial)	Phase II RCT (NCT03919461)	Resectable CRC (Stage I-III)	200 (Not specified)	PrOH (non-selective β_1_/β_2_); dose not specified	20 days perioperative	Etodolac, dose not specified	Ongoing trial; Recruitment completed; results pending	Ongoing trial; results not yet available	[147]

**Abbreviations:** β-blockers—β-adrenergic receptor blockers; β_1_—beta-1 adrenergic receptor; β_2_—beta-2 adrenergic receptor; CI—confidence interval; COMPIT—Combined Metabolic and Perioperative Intervention Trial; CRC—colorectal cancer; HR—hazard ratio; mCRC—metastatic colorectal cancer; n—number of patients in the β-blocker treatment group; OS—overall survival; PFS—progression-free survival; PrOH—propranolol; RCT—randomized controlled trial; TME—tumor microenvironment. Clinical outcomes reported in observational studies represent adjusted associations and should not be interpreted as evidence of causality. Differences in β-blocker selectivity, dose, treatment duration, concomitant therapies, and study design limit direct comparison across studies.

**Table 4:** Representative preclinical and clinical studies evaluating β-blocker dosing in colorectal cancer compared with clinically approved human dosing regimens.

Study	Study Type	β-Blocker	Reported Dose/Concentration	Approved Human Therapeutic Dose/Exposure	Translational Relevance	References
Puzderova et al. (2023)	In vitro	PrOH	50 μM	Therapeutic plasma concentrations are substantially lower	Concentration exceeds clinically achievable plasma exposure	[91]
Barathova et al. (2020)	In vitro	PrOH	50 μM	Therapeutic plasma concentrations are substantially lower	Concentration exceeds clinically achievable plasma exposure	[62]
Alzahrani et al. (2025)	In vitro	PrOH	2–320 µM	Therapeutic plasma concentrations are substantially lower	Only the lowest concentrations approach clinically relevant exposure; higher concentrations exceed clinically achievable plasma levels	[121]
Qiao et al. (2021)	In vivo (mouse model)	PrOH	200 μg	Approved human dose: 40–320 mg/day	Dose is higher than routinely prescribed cardiovascular dosing after interspecies dose conversion	[138,163]
Fjæstad et al. (2022)	In vivo (mouse model)	PrOH	50–100 mg/kg/day	Approved human dose: 40–320 mg/day	Dose substantially exceeds approved human therapeutic dosing	[93,163]
Anselmino et al. (2023)	Combined in vitro/in vivo (mouse model)	PrOH	2.5 µM 7 mg/kg/day	Approved human dose: 40–320 mg/day	In vitro concentration is clinically relevant, whereas the in vivo dose exceeds approved human dosing	[154,163]
Hu et al. (2021)	In vivo (mouse model)	PrOH	2 mg/kg/week	Approved human dose: 40–320 mg/day	Dose falls within or close to the clinically relevant exposure range	[155,163]
Fiala et al. (2019)	Retrospective cohort study	Cardioselective and non-selective β-blockers	Standard cardiovascular doses	Standard approved cardiovascular dosing	Routine clinical dosing; exact dose not reported	[157]
Haldar et al. (2020)	Phase II randomized, double-blind, placebo-controlled biomarker RCT	PrOH	20–80 mg twice daily (40–160 mg/day)	Approved human dose: 40–320 mg/day	Dose falls within the approved therapeutic range	[53,163]
Ahl et al. (2019)	Nationwide observational cohort study	Predominantly β1-selective β-blockers	Standard cardiovascular doses	Standard approved cardiovascular dosing	Routine clinical dosing; exact dose not reported	[159]
Ricon-Becker et al. (2023)	Long-term follow-up of a Phase II randomized controlled trial	PrOH	20–80 mg twice daily (40–160 mg/day)	Approved human dose: 40–320 mg/day	Dose falls within the approved therapeutic range	[146,163]
Emilsson et al. (2025)	Emulated target trial (nationwide retrospective cohort)	Unspecified	Not specified	Not reported	Dose information unavailable	[162]
Sakis et al. (2026) (COMPIT1 trial)	Ongoing Phase II randomized controlled trial	PrOH	Not specified	Not reported	Dose information not yet available	[147]

**Abbreviations:** PrOH—propranolol; RCT—randomized controlled trial. Note: Therapeutic human doses refer to approved cardiovascular indications. Mouse doses are presented as reported in the original studies and should not be directly equated with human doses. The assessment of translational relevance is based on approximate pharmacological comparability and should be interpreted cautiously because of interspecies differences in pharmacokinetics and pharmacodynamics.

## Data Availability

No new data were created or analyzed in this study. Data sharing is not applicable to this article.

## Abbreviations

The following abbreviations are used in this manuscript:

β-AR	β-Adrenergic Receptor
Ang-1	Angiopoietin-1
Ang-2	Angiopoietin-2
APC	Adenomatous Polyposis Coli
ARs	Adrenergic Receptors
ATF	Activating Transcription Factor
BCL-2	B-Cell Lymphoma 2
CAFs	Cancer-Associated Fibroblasts
CAIX	Carbonic Anhydrase IX
cAMP	Cyclic Adenosine Monophosphate
CAP	Capecitabine
CAR-NK	Chimeric Antigen Receptor Natural Killer Cell Therapy
CAR-T	Chimeric Antigen Receptor T-Cell Therapy
CIMP	CpG island methylation phenotype
CIN	Chromosomal Instability
CMS	Consensus Molecular Subtype
COX-2	Cyclooxygenase-2
CRC	Colorectal Cancer
CRE	cAMP Response Element
CREB	cAMP-Responsive Element Binding Protein
ctDNA	Circulating Tumor DNA
CTLA-4	Cytotoxic T-Lymphocyte-Associated Protein 4
Cu-PN	Copper-Propranolol Nanoparticles
CQ	Chloroquine
DAMPs	Damage-Associated Molecular Patterns
DCs	Dendritic Cells
DFS	Disease-Free Survival
dMMR	Deficient Mismatch Repair
DNA	Deoxyribonucleic Acid
ECM	Extracellular Matrix
EGFR	Epidermal Growth Factor Receptor
Elk 1	ETS-Like Protein 1
EMT	Epithelial–Mesenchymal Transition
Epi	Epinephrine
ERK1/2	Extracellular Signal-Regulated Kinases 1 and 2
GDP	Guanosine Diphosphate
GPCRs	G Protein-Coupled Receptors
GRKs	G Protein-Coupled Receptor Kinases
GSDME	Gasdermin E
GTP	Guanosine Triphosphate
HER2	Human Epidermal Growth Factor Receptor 2
HIF-1α	Hypoxia-Inducible Factor 1-Alpha
ICD	Immunogenic Cell Death
ICIs	Immune Checkpoint Inhibitors
IDO	Indoleamine 2,3-Dioxygenase
IFN-γ+	Interferon-Gamma-Positive
IL-6	Interleukin-6
IL-10	Interleukin-10
mCRC	Metastatic Colorectal Cancer
MDSCs	Myeloid-Derived Suppressor Cells
MEK1/2	Mitogen-Activated Protein Kinase 1/2
MLKL	Mixed Lineage Kinase Domain-Like Protein
MMPs	Matrix Metalloproteinases
MMP-2	Matrix Metalloproteinase 2
MSI	Microsatellite Instability
MSI-H/dMMR	High Microsatellite Instability/Deficient Mismatch Repair
MSS	Microsatellite Stable
MSS/pMMR	Microsatellite Stable/Proficient Mismatch Repair
NE	Norepinephrine
NK	Natural Killer
OS	Overall Survival
pAKT	Phosphorylated AKT
PDEs	Phosphodiesterases
PDGF	Platelet-Derived Growth Factor
PD-L1	Programmed Death-Ligand 1
PD-1	Programmed Cell Death Protein 1
pERK	Phosphorylated ERK
PFS	Progression-Free Survival
PI3Ks	Phosphoinositide 3-Kinases
PI3K/Akt	Phosphoinositide 3-Kinase/Protein Kinase B
PKA	Protein Kinase A
PKC	Protein Kinase C
pMEK	Phosphorylated MEK
PrOH	Propranolol
PTEN	Phosphatase and Tensin Homolog
RIPK1	Receptor-Interacting Protein Kinase 1
RIPK3	Receptor-Interacting Protein Kinase 3
ROS	Reactive Oxygen Species
RTKs	Receptor Tyrosine Kinase
TAMs	Tumor-Associated Macrophages
TCR	TCR-Engineered T Cells
TGF-β	Transforming Growth Factor-Beta
Th2	T Helper 2 Cells
TILs	Tumor-Infiltrating Lymphocytes
TME	Tumor Microenvironment
Tregs	Regulatory T-Cells
VEGF	Vascular Endothelial Growth Factor
2D	Two-Dimensional
3D	Three-Dimensional
5-FU	5-Fluorouracil
